# Nonalcoholic steatohepatitis medical patient journey from the perspective of hepatologists, gastroenterologists and patients: a cross-sectional survey

**DOI:** 10.1186/s12876-022-02410-x

**Published:** 2022-07-10

**Authors:** Mary Rinella, Donna R. Cryer, Amy Articolo, Travis Fisher, Jennifer Schneider, Karl Nadolsky

**Affiliations:** 1 Department of Medicine, Section of Gastroenterology, Hepatology and Nutrition, University of Chicago Pritzker School of Medicine, Chicago, IL USA; 2 Global Liver Institute, Washington, DC USA; 3 Novo Nordisk Inc, Plainsboro, NJ USA; 4 Department of Medicine, Michigan State University College of Human Medicine, Grand Rapids, MI USA

**Keywords:** Nonalcoholic fatty liver disease, Diabetes mellitus, type 2, Obesity, Self report

## Abstract

**Background:**

Nonalcoholic steatohepatitis (NASH), the inflammatory subtype of nonalcoholic fatty liver disease, is underdiagnosed and expected to become the leading indication for liver transplant in the United States. We aimed to understand the medical journey of patients with NASH and role of hepatologists/gastroenterologists in diagnosing and treating patients with NASH.

**Methods:**

A United States population-based cross-sectional online survey was completed by 226 healthcare professionals (HCPs) who treat patients with NASH and 152 patients with NASH; this study focuses on the patient and 75 hepatologist/gastroenterologist HCP respondents. Tests of differences (chi square, t-tests) between respondent types were performed using SPSS.

**Results:**

Most patients reported receiving their diagnosis of NASH from a hepatologist (37%) or gastroenterologist (26%). Hepatologists/gastroenterologists were more likely than other HCPs to use FibroScan (transient elastography) to diagnose NASH and were more likely to distinguish between NASH with or without fibrosis. Hepatologists/gastroenterologists (68%) and patients (52%) agree that hepatologists/gastroenterologists are the primary coordinators of NASH care. The majority of hepatologists/gastroenterologists (85%) are aware of American Association for the Study of Liver Diseases (AASLD) clinical practice guidance, and 86% of those aware consider them when diagnosing patients with NASH. Hepatologists/gastroenterologists most frequently recommended exercise (86%), diet (70%), and supplements (58%) for ongoing management of NASH. Pharmaceutical medications for comorbidities were prescribed by a minority of hepatologists/gastroenterologists for their patients with NASH. Hepatologists/gastroenterologists cite difficulty (67%) or unwillingness (64%) to adhere to lifestyle changes as primary reasons patients with NASH discontinue NASH treatment.

**Conclusions:**

Hepatologists/gastroenterologists are considered the coordinators of NASH care. While recognizing that patient adherence to lifestyle changes is the basis for successful treatment, important barriers limit successful implementation.

**Supplementary Information:**

The online version contains supplementary material available at 10.1186/s12876-022-02410-x.

## Background

Nonalcoholic steatohepatitis (NASH) is the inflammatory form of nonalcoholic fatty liver disease (NAFLD). Both NAFLD and NASH are characterized by identification of > 5% hepatic steatosis in the absence of excessive alcohol consumption and other etiologies [1]. NASH is characterized by steatosis accompanied by hepatocyte inflammation and injury (ballooning) [1], which can perpetuate the development of fibrosis. The extent of liver fibrosis is the greatest predictor of hepatic morbidity and mortality [2–6]. Both NAFLD and NASH can develop progressive hepatic fibrosis, though this is more likely in the context of NASH [7–9].

Globally, 25% of people are estimated to have NAFLD and 2–6% are estimated to have NASH [10]. In the US, NAFLD prevalence is estimated at 37%; among patients with NAFLD, approximately 8% have advanced liver fibrosis [11]. The prevalence of NASH is increasing, but more concerning is the disproportionate increase in those with advanced fibrosis, hepatocellular carcinoma, and hepatic decompensation predicted by modeling studies [12]. In the United States, NASH is currently the leading indication for liver transplant in women and those > 65 years of age and is predicted to become the overall leading indication for liver transplant [13]. While there are currently no FDA approved pharmacologic treatments for NASH [14], management focused on optimal control of comorbidities, using available therapies that may have additive benefit in NASH, as well as implementation and sustained support for lifestyle change can improve liver health and overall health in patients with NASH [14].

NASH is underdiagnosed as patients are often asymptomatic or present with non-specific symptoms [15]. According to 2018 American Association for the Study of Liver Diseases (AASLD) guidance, NAFLD can be diagnosed by imaging or liver biopsy while NASH requires a liver biopsy [1]. Many patients and healthcare professionals (HCPs) are hesitant to use liver biopsies due to costs and associated risks [16]. The requirement for liver biopsy to confirm diagnosis could be contributing to the under-diagnosis of NASH; liver biopsies are potentially dangerous, resource-intensive, and have limited scalability [17]. The field is looking to non-invasive tests (NITs) to identify those at most risk, who can then be targeted for therapy or more confidently exclude those who do not need specialty care [18]. Noninvasive risk stratification algorithms using calculations (e.g., Fibrosis-4 Index [FIB4]) followed by liver stiffness measurements in those with type 2 diabetes mellitus or high risk, are being proposed and refined to aid HCPs in their management [18, 19].

This study seeks to understand the medical journeys of patients with NASH, the role of hepatologists and gastroenterologists in diagnosing and treating patients with NASH, and to identify areas for improvement in the diagnosis and treatment of NASH in the United States (US).

## Methods

### Study design and participants

A cross-sectional online survey was conducted among patients diagnosed with NASH and healthcare professionals (HCPs) treating patients with NASH. Data were collected from November 10th, 2020 to January 1st, 2021. All respondents were recruited via email through online panel companies. Respondents provided permission to be contacted for research purposes. Eligible participants completing the entire survey received a modest monetary incentive.

Survey questions were informed by a literature review and qualitative interviews which were conducted with patients with NASH and HCPs. Separate surveys were used for each audience to measure attitudes and experiences with NASH from before diagnosis to treatment. The surveys consisted of a variety of yes/no, multiple-choice, and Likert-scale questions (See Additional file 1: HCP Survey and Additional file 2: Patient Survey, which demonstrate the surveys used). The study samples were independent; patients and HCPs surveyed were not matched pairs.

All methods were carried out in accordance with relevant guidelines and regulations. The surveys were conducted in accordance with the principles and guidelines established by the Office for Human Research Protections, the Insights Association Code of Standards and Ethics, and the Declaration of Helsinki. The study was reviewed by the Western Institutional Review Board and was determined to qualify for exempt status. Respondents reviewed information about the purpose and nature of the survey. Respondents selected a yes/no option indicating informed consent to participate in the study prior to entering the screening portion of the survey. If they consented, they continued to the survey questions. Respondents could discontinue the survey at any time.

Patients included were US residents, age ≥ 18 years, diagnosed with NASH within the past 10 years, currently seeing an HCP to treat and manage NASH, and reported awareness of diagnostic screens completed. HCPs included were employed in US facilities (except Maine and Vermont to comply with Sunshine reporting requirements); physicians practicing as primary care physicians, gastroenterologists, hepatologists, or endocrinologists; treated at least five patients (primary care physicians) or 20 patients (gastroenterologists, hepatologists, endocrinologists) in the past month with NASH; had board certification or eligibility in their chosen specialty; were in practice for 3–25 years; and were not based in a government facility or an ambulatory surgical center.

### Statistical analyses

We performed descriptive statistical analysis (means, frequencies) using SPSS Statistics for Windows 23 (SPSS, Chicago, Illinois). Tests of differences (chi square, t-tests) within respondent types were performed using SPSS. Statistical significance was set at p < 0.05, using 2-tailed tests. The final patient sample was weighted to representative racial demographic targets for the US NASH population derived from published literature [20]. HCP data were not weighted. All reported statistics are weighted accordingly with the exception of demographic data which are reported unweighted as a means to characterize the raw sample data (Table 1).

**Table 1:** Sample characteristics

Characteristics of survey respondents		Patients with NASH (n = 152)
Sex, *n* (%)		
Male		94 (62)
Female		58 (38)
Mean age (SD), *years*		40 (11)
Region, *n* (%)		
Northeast		33 (22)
Midwest		24 (16)
South		44 (29)
West		51 (34)
Ethnicity, *n* (%)		
White		62 (41)
Black/African American		35 (23)
Spanish/Hispanic/Latino		45 (30)
Other		10 (7)

	Healthcare professionals (n = 226)	Hepatologists/gastroenterologists (n = 75)
Sex, *n* (%)		
Male	169 (75)	61 (81)
Female	54 (24)	13 (17)
Other	3 (1)	1 (1)
Mean time in practice, *years (SD)*	19 (7)	18 (7)
Region, *n* (%)		
Northeast	64 (28)	25 (33)
Midwest	53 (23)	18 (24)
South	71 (31)	23 (31)
West	38 (17)	9 (12)
Professional specialty, *n* (%)		
PCP	101 (45)	
Hepatologist/gastroenterologist	75 (33)	
Endocrinologist	50 (22)	
Gastroenterologist		64 (85)
Hepatologist		11 (15)

*Data are mean (SD) or number (%) and are reported for the final unweighted sample

*NASH*, nonalcoholic steatohepatitis; *PCP*, primary care physician; *SD*, standard deviation

## Results

### Sample characteristics

A total of 152 patients with NASH and 226 HCPs (101 primary care physicians (PCPs), 75 gastroenterologists/hepatologists, 50 endocrinologists) completed the survey (Table 1). This manuscript focuses on responses from patients and the 75 gastroenterologists and hepatologists surveyed.

### NASH diagnosis

**Fig. 1:**
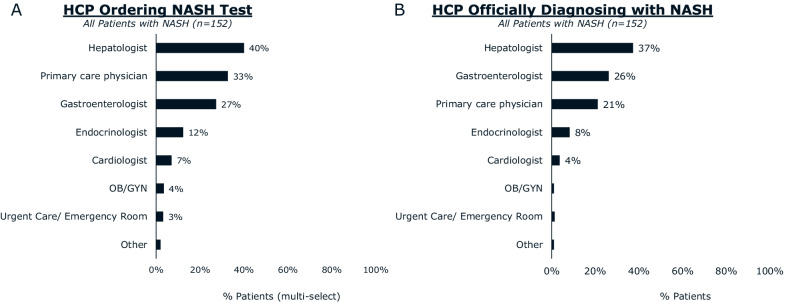
HCPs ordering NASH diagnostic tests and diagnosing patients with NASH. According to patients with NASH, **A** ordering of NASH diagnostic tests and **B** determination of NASH diagnosis was most commonly performed by hepatologists. Abbreviations: HCP, healthcare professional; NASH, nonalcoholic steatohepatitis; OB/GYN, obstetrician/gynecologist

**Fig. 2:**
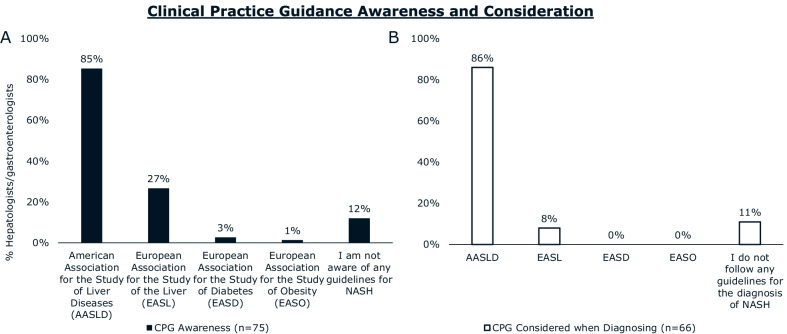
Clinical practice guidance awareness and consideration during diagnosis. Proportion of hepatologists and gastroenterologists reporting they are **A** aware of clinical practice guidance for NASH and **B** consider them when diagnosing. Only physicians who reported awareness of clinical practice guidance were asked whether they consider them during diagnosis. Abbreviations: CPG, clinical practice guidance

Over two thirds of patients reported hepatologists (40%) and gastroenterologists (27%) ordered the tests that led to a formal diagnosis of NASH. The majority of patients received their official NASH diagnosis from a hepatologist (37%) or a gastroenterologist (26%) (Fig. 1). Most hepatologists and gastroenterologists (85%) say they are aware of AASLD clinical practice guidance for NASH. Of those who are aware of guidance, 86% say they consider them when diagnosing patients with NASH (Fig. 2).

**Fig. 3:**
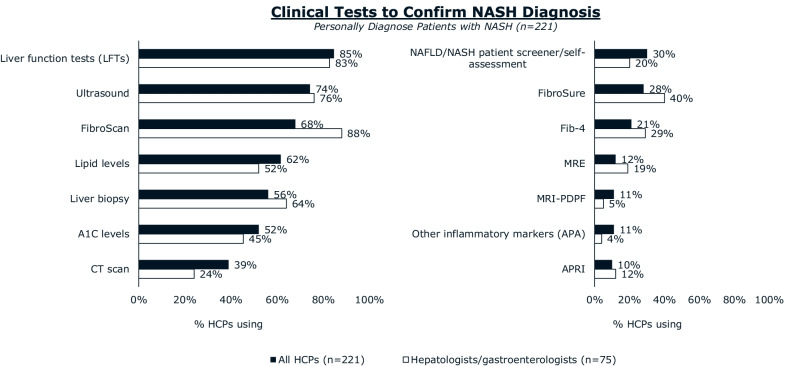
Clinical tests used to confirm NASH diagnosis. Tests used for NASH diagnosis were reported by HCPs who personally diagnose patients with NASH. Abbreviations: A1C, hemoglobin A1C; APA, anti-phospholipid antibodies; APRI, AST to Platelet Ratio Index; CT, computed tomography; Fib-4, Fibrosis-4 Index; HCPs, healthcare professionals; MRI-PDFF, magnetic resonance imaging derived proton density fat fraction; MRE, magnetic resonance elastography; NASH, nonalcoholic steatohepatitis

The tests most commonly used by hepatologists and gastroenterologists to confirm NASH diagnosis included FibroScan (transient elastography) (Echosens, Paris, France) (88%), liver function tests (83%), ultrasound (76%), and liver biopsy (64%). Hepatologists and gastroenterologists were more likely than other HCPs to use transient elastography while diagnosing NASH (Fig. 3). Less than half of hepatologists and gastroenterologists reporting using FibroSure (Laboratory Corporation of America, Raritan, NJ, US) (40%), FIB-4 scores (29%), or magnetic resonance elastography (19%) to confirm NASH diagnosis (Fig. 3). Other HCPs were even less likely to use these tools to confirm NASH diagnosis.

Almost all (89%) hepatologists and gastroenterologists reported that they distinguish between NASH with or without fibrosis. This was significantly higher than the proportion of PCPs (60%) and endocrinologists (67%) who distinguish between NASH with and without fibrosis. A plurality of patients with NASH (44%) reported that their formal NASH diagnosis was characterized simply as NASH. Less than half of patients said they received a diagnosis that mentioned their fibrosis state: 33% reporting NASH with fibrosis and 5% without fibrosis. Only 53% of patients with NASH reported receiving a liver biopsy to confirm their NASH diagnosis.

Patients with NASH reported that diagnosis discussions included causes of NASH (62%), NASH’s impact on other conditions (53%), and treatments for NASH (53%). Hepatologists and gastroenterologists most commonly reported discussing NASH treatments (85%), progression to cirrhosis (81%), progression to fibrosis (80%), and cause of NASH (80%). Only 29% of patients said that progression of NASH leading to a liver transplant was covered in diagnosis discussions.

### Hepatologist and gastroenterologist role in managing NASH

**Fig. 4:**
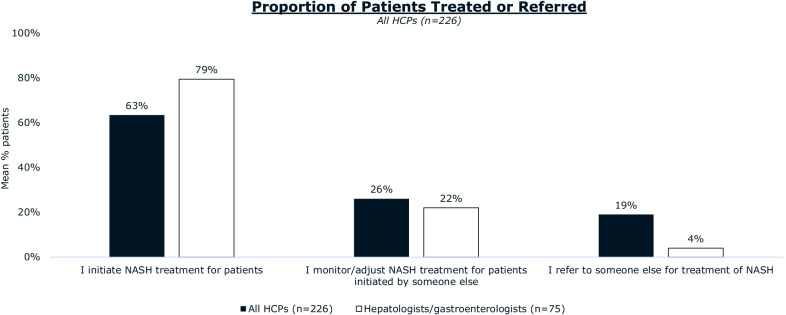
Proportion of patients with NASH treated/referred expressed as mean percentage estimates reported by HCPs. Hepatologists and gastroenterologists estimate that they treat most of their patients with NASH themselves. Abbreviations: HCP, healthcare professional; NASH, nonalcoholic steatohepatitis

Most patients (83%) in our study had initial discussions about symptoms with an HCP, followed by a NASH diagnosis, and, finally, initial treatment for their NASH; we refer to this as the most common patient journey. In the most common patient journey, just over half of patients with NASH reported receiving their initial NASH treatment from hepatologists and gastroenterologists. Hepatologists and gastroenterologists estimated that they initiate NASH treatment (79%) or monitored/adjusted NASH treatments initiated by other healthcare professionals (22%) for almost all of their patients (Fig. 4). Hepatologists and gastroenterologists estimated that they only refer 4% of patients with NASH to other healthcare professionals for treatment.

**Fig. 5:**
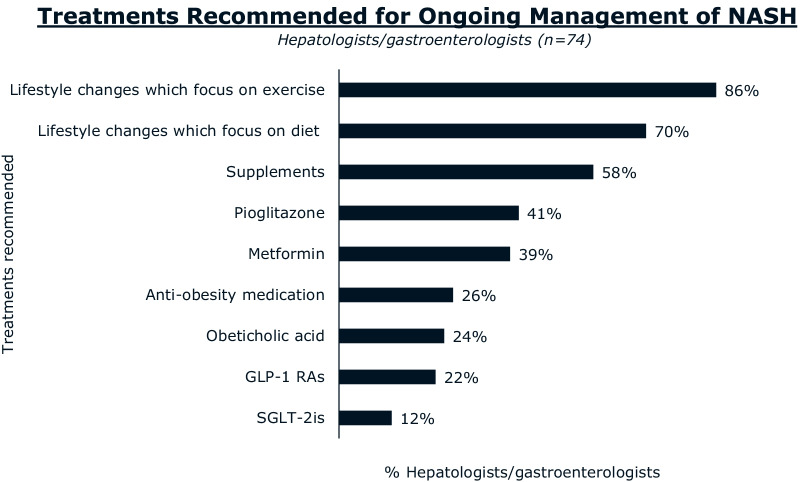
Hepatologists and gastroenterologists reporting that they personally recommend treatments for the ongoing management of NASH. Abbreviations: GLP-1 RAs, glucagon-like peptide-1 receptor agonists; NASH, nonalcoholic steatohepatitis; SGLT-2is, sodium glucose cotransporter-2 inhibitors

Lifestyle and diet changes were the most commonly recommended NASH treatments and medications were the least frequently recommended (Fig. 5). Hepatologists and gastroenterologists responded that they most frequently recommended exercise (86%), diet (70%), and supplements (58%) for ongoing management of NASH. Fewer hepatologists and gastroenterologists prescribed anti-obesity medications and diabetes medications such as pioglitazone (41%), metformin (39%), GLP-1 receptor agonists (22%), and SGLT-2 inhibitors (12%) (Fig. 5).

**Fig. 6:**
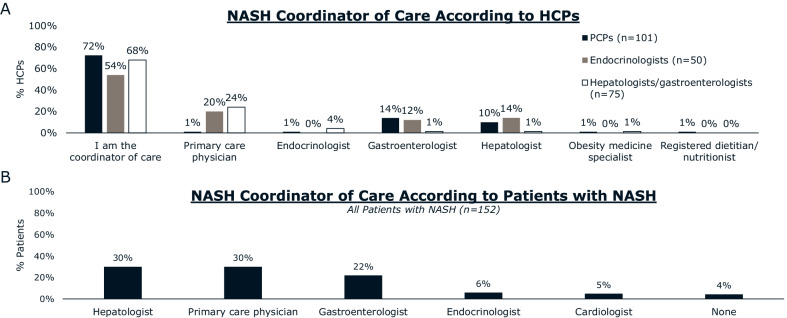
The coordinator of NASH care according to **A** HCPs and **B** patients with NASH. Abbreviations: HCP, healthcare professional; NASH, nonalcoholic steatohepatitis; PCP, primary care physician

**Fig. 7:**
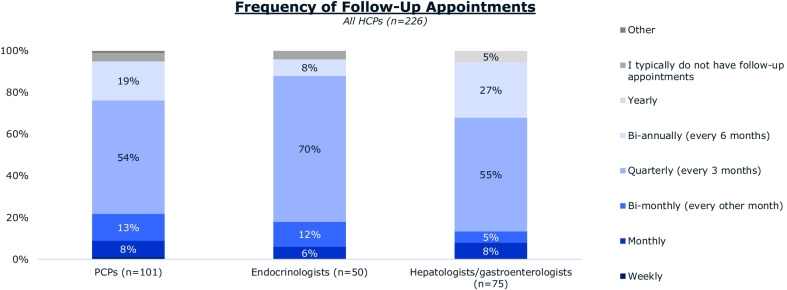
Frequency of NASH follow-up appointments following NASH diagnosis according to PCPs, endocrinologists, hepatologists and gastroenterologists. Abbreviations: HCP, healthcare professional; PCP, primary care physician

The majority of hepatologists and gastroenterologists (68%) and patients (52%) agree that hepatologists and/or gastroenterologists are the primary coordinators of NASH care (Fig. 6). Most hepatologists and gastroenterologists (55%) reported having quarterly follow-up appointments with patients with NASH after diagnosis; 13% scheduled monthly/bi-monthly appointments and 32% scheduled bi-annually or yearly follow-up appointments (Fig. 7).

### Behaviors and perceptions

Hepatologists and gastroenterologists cite difficulty with lifestyle changes (67%) or lack of adherence to lifestyle changes (64%) as the primary reasons patients with NASH discontinue treatment. Among a small subset of patients with NASH (n = 15) who had seen multiple HCPs for comorbidities, only 11% said a hepatologist had been the most helpful with lifestyle management compared to 38% selecting gastroenterologists. In this same group of patients with NASH, 44% considered PCPs the most helpful with lifestyle management. The reasons patients found certain HCPs more helpful in supporting lifestyle change included frequent visits, positive encouragement, and informative advice. When asked what types of support would be most helpful to patients with NASH, more than half of PCPs (58%) and endocrinologists (60%) mentioned access to a physician who specialized in NASH. Most patients (62%) agreed that access to a physician who specialized in NASH would be one of the most helpful types of support.

## Discussion

NASH is a growing problem and will likely be the primary cause of liver transplant in the near future [13]. To meet the needs of this growing patient population and curb the increase, awareness must be raised, diagnosis rates increased, and treatments improved. Across the different groups of HCPs we queried, the majority within each group view themselves as the coordinator of care for patients with NASH suggesting that the clinical roles and responsibilities are unclear, but the majority are willing to take on the challenge of managing NASH. Hepatologists and gastroenterologists can take leadership in defining care pathways.

Most patients reported that hepatologists and gastroenterologists conducted the tests that led to diagnosis and officially diagnosed them with NASH. Patients and other HCPs want a physician specializing in NASH to support treatment. Patients with more severe fibrosis have an increased risk of morbidity and mortality [2, 3]. Quantification of hepatic fibrosis provides an indication of current risk level and can inform the treatment plan of patients with NASH [18, 19]. The extent of fibrosis is an important metric of NASH improvement or worsening. Hepatologists and gastroenterologists were more likely than other HCPs to distinguish between NASH with and without fibrosis. This may reflect a better understanding of the disease or greater access to tools for the evaluation of fibrosis.

Hepatologists and gastroenterologists should work to improve communication of test results to patients. In our study many patients with NASH (44%) reported that they received diagnoses of NASH without qualifiers (e.g., a diagnosis of NASH rather than NASH with moderate/severe fibrosis). This suggests that a key role for hepatologists and gastroenterologists is to define disease progression and prognosis more specifically and that the stage of a patient’s liver disease may inform their treatment strategy.

Hepatologists and gastroenterologists can use their expertise in clinical practice guidance and knowledge of the importance of fibrosis evaluation to lead/guide care for patients with NASH. Hepatologists and gastroenterologists are leading efforts to modify existing guidance and develop new guidance for primary care to support initial diagnosis and referrals to specialists. New AASLD and AGA guidance emphasizes the importance of algorithmic diagnostic pathways that emphasize NITs and reserve liver biopsies for high-risk patients where NITs have proved inconclusive [18, 19]. There is room to use NITs such as FIB-4 derived from routine clinical variables (e.g. age, blood tests etc.) to assist in the identification of fibrotic NASH [15]. Transient elastography is a relatively inexpensive NIT and our study shows that it is already highly used by hepatologists and gastroenterologists [21].

Hepatologists and gastroenterologists face a number of challenges in diagnosing and treating NASH. NASH is underdiagnosed [22]; the current requirement for liver biopsy to diagnose NASH may be a barrier to more frequent NASH diagnosis [17]. Although a liver biopsy is the reference standard for the diagnosis of NASH [1], many of the patients in our study (47%) said they did not receive a biopsy to establish their NASH diagnosis. Determining when and where the risk associated with a biopsy is deemed appropriate is largely a judgment call, though several algorithms have been proposed [18, 23]. Work remains to be done to educate all HCPs on the appropriate method of risk stratification in the context of NAFLD.

NASH is a chronic condition that requires regular monitoring and management [24]. Frequent follow-ups and a multi-pronged approach are important for managing these diseases [25]. In this study, hepatologists and gastroenterologists had the lowest frequency of follow-up appointments suggesting more frequent touchpoints with patients might require a different care management paradigm. A longitudinal study of Japanese patients with NAFLD found that use of insulin and decrease in hemoglobin A1C levels were significantly associated with reductions in liver fibrosis [26]. Similarly, weight loss achieved with anti-obesity medications [27], lifestyle changes [28], or bariatric surgery [29] can have a positive impact on NASH. Inexperience with anti-obesity medications may be preventing hepatologists and gastroenterologists from utilizing all available resources to treat NASH [27]. Experts believe that a multidisciplinary approach involving dietitians, behavioral therapists, and physical activity supervisors is important to successful NAFLD/NASH management [30]. The aim is to improve the effectiveness of lifestyle change by improving the counselling and advice patients receive, providing actionable diet and weight loss strategies, and supporting patients as they attempt to make lifestyle changes.

Survey research provides a unique perspective on how patients and HCPs interact with the healthcare system but comes with limitations. Self-reported experiences may reflect inaccurate or incomplete recollection on the part of survey participants [31]. The groups of HCPs and patients with NASH we surveyed are unpaired: the HCPs were not matched with patients they were treating. In some instances, HCPs and patients appear to disagree about the frequency with which certain events occur (e.g., tests conducted to diagnose NASH). Sometimes these differences may reflect differences in perception between HCPs and patients. Other times, these differences may reflect real differences in the HCP and patient populations sampled. The patients and physicians who responded to this survey may differ in important ways from those who did not respond to this survey; this could limit the generalizability of these findings to the wider population of patients with NASH and physicians treating NASH in the US.

Hepatologists and gastroenterologists have unique clinical roles, which have not been explored in this study. This study involved a total of 75 respondents for these specialties that included 64 gastroenterologists and 11 hepatologists. Hepatologists and gastroenterologists were grouped to provide meaningful numbers for evaluation. However, grouping gastroenterologists and hepatologists together limits our ability to provide insights for each specialty and may be hiding important differences between the two types of specialists and how they manage patients with NASH.

## Conclusions

Hepatologists and gastroenterologists can leverage their roles as coordinators of care for patients with NASH to encourage better practices for diagnosis and treatment. The diagnostic process is highly variable; many different diagnostic tests are used to confirm NASH diagnosis. There is room for improvement and standardization in NASH diagnosis. Utilization of NITs and risk stratification algorithms may lead to diagnosis of more patients with NASH and at earlier stages of disease and new guidance from professional medical societies represents a step in the right direction by advocating increased use of NITs. Hepatologists and gastroenterologists can advocate greater use of medications for diabetes, obesity, and other comorbidities where appropriate to tackle the growing problem of NASH. Hepatologists and gastroenterologists should embrace a multidisciplinary approach to NASH treatment and national guidance should be updated to clearly define roles and responsibilities of various clinicians [18, 32].

## Supplementary Information

**Additional file 1**. NASH_hepgastro_Additional file 1, HCP Survey.pdf HCP Survey, survey used to collect responses from healthcare professionals

**Additional file 2**. NASH_hepgastro_Additional file 2, Patient Survey.pdf Patient Survey, survey used to collect responses from patients with NASH

## Data Availability

The datasets generated during the current study are not publicly available due to containing proprietary information but are available from the corresponding author on reasonable request.

## Abbreviations

AASLD: American Association for the Study of Liver Diseases
AGA: American Gastroenterological Association
CPG: Clinical practice guidance
CT: Computed tomography
FDA: Food and Drug Administration
FIB4: Fibrosis-4 Index
HCP: Healthcare professional
MRE: Magnetic resonance elastography
NAFLD: Nonalcoholic fatty liver disease
NASH: Nonalcoholic steatohepatitis
NIT: Non-invasive tests
PCP: Primary care physician
SD: Standard deviation
US: United States

## References

[1] Chalasani N, Younossi Z, Lavine JE, Charlton M, Cusi K, Rinella M (2018). The diagnosis and management of nonalcoholic fatty liver disease: practice guidance from the American association for the study of liver diseases. Hepatology.

[2] Vilar-Gomez E, Calzadilla-Bertot L, Wai-Sun Wong V, Castellanos M, Aller-de la Fuente R, Metwally M (2018). Fibrosis severity as a determinant of cause-specific mortality in patients with advanced nonalcoholic fatty liver disease: a multi-national cohort study. Gastroenterology.

[3] Dulai PS, Singh S, Patel J, Soni M, Prokop LJ, Younossi Z (2017). Increased risk of mortality by fibrosis stage in nonalcoholic fatty liver disease: systematic review and meta-analysis. Hepatology.

[4] Younossi ZM, Stepanova M, Rafiq N, Makhlouf H, Younoszai Z, Agrawal R (2011). Pathologic criteria for nonalcoholic steatohepatitis: interprotocol agreement and ability to predict liver-related mortality. Hepatology.

[5] Ekstedt M, Hagström H, Nasr P, Fredrikson M, Stål P, Kechagias S (2015). Fibrosis stage is the strongest predictor for disease-specific mortality in NAFLD after up to 33 years of follow-up. Hepatology.

[6] Angulo P, Kleiner DE, Dam-Larsen S, Adams LA, Bjornsson ES, Charatcharoenwitthaya P (2015). Liver fibrosis, but no other histologic features, is associated with long-term outcomes of patients with nonalcoholic fatty liver disease. Gastroenterology.

[7] Adams LA, Sanderson S, Lindor KD, Angulo P (2005). The histological course of nonalcoholic fatty liver disease: a longitudinal study of 103 patients with sequential liver biopsies. J Hepatol.

[8] Fassio E, Alvarez E, Domínguez N, Landeira G, Longo C (2004). Natural history of nonalcoholic steatohepatitis: a longitudinal study of repeat liver biopsies. Hepatology.

[9] Wong VW, Wong GL, Choi PC, Chan AW, Li MK, Chan HY (2010). Disease progression of non-alcoholic fatty liver disease: a prospective study with paired liver biopsies at 3 years. Gut.

[10] Younossi ZM, Koenig AB, Abdelatif D, Fazel Y, Henry L, Wymer M (2016). Global epidemiology of nonalcoholic fatty liver disease-Meta-analytic assessment of prevalence, incidence, and outcomes. Hepatology.

[11] Ciardullo S, Perseghin G (2021). Prevalence of NAFLD, MAFLD and associated advanced fibrosis in the contemporary United States population. Liver Int.

[12] Estes C, Anstee QM, Arias-Loste MT, Bantel H, Bellentani S, Caballeria J (2018). Modeling NAFLD disease burden in China, France, Germany, Italy, Japan, Spain, United Kingdom, and United States for the period 2016–2030. J Hepatol.

[13] Noureddin M, Vipani A, Bresee C, Todo T, Kim IK, Alkhouri N (2018). NASH leading cause of liver transplant in women: updated analysis of indications for liver transplant and ethnic and gender variances. Am J Gastroenterol.

[14] Sumida Y, Yoneda M (2018). Current and future pharmacological therapies for NAFLD/NASH. J Gastroenterol.

[15] Sheka AC, Adeyi O, Thompson J, Hameed B, Crawford PA, Ikramuddin S (2020). Nonalcoholic steatohepatitis: a review. JAMA.

[16] Nalbantoglu IL, Brunt EM (2014). Role of liver biopsy in nonalcoholic fatty liver disease. World J Gastroenterol.

[17] Deemer J, Heinz S (2019). PDB84 liver biopsy—a bottleneck to nash diagnosis. Value Health.

[18] Kanwal F, Shubrook JH, Younossi Z, Natarajan Y, Bugianesi E, Rinella ME (2021). Preparing for the NASH epidemic: a call to action. Gastroenterology.

[19] Ando Y, Jou JH (2021). Nonalcoholic fatty liver disease and recent guideline updates. Clin Liver Dis.

[20] Rich NE, Oji S, Mufti AR, Browning JD, Parikh ND, Odewole M (2018). Racial and ethnic disparities in nonalcoholic fatty liver disease prevalence, severity, and outcomes in the United States: a systematic review and meta-analysis. Clin Gastroenterol Hepatol.

[21] Hashemi SA, Alavian SM, Gholami-Fesharaki M (2016). Assessment of transient elastography (FibroScan) for diagnosis of fibrosis in non-alcoholic fatty liver disease: a systematic review and meta-analysis. Caspian J Intern Med.

[22] Rinella ME, Lominadze Z, Loomba R, Charlton M, Neuschwander-Tetri BA, Caldwell SH (2016). Practice patterns in NAFLD and NASH: real life differs from published guidelines. Therap Adv Gastroenterol.

[23] Cotter TG, Rinella M (2020). Nonalcoholic fatty liver disease 2020: the state of the disease. Gastroenterology.

[24] Cusi K (2020). Time to include nonalcoholic steatohepatitis in the management of patients with type 2 diabetes. Diabetes Care.

[25] Turer CB (2015). Tools for successful weight management in primary care. Am J Med Sci.

[26] Hamaguchi E, Takamura T, Sakurai M, Mizukoshi E, Zen Y, Takeshita Y (2010). Histological course of nonalcoholic fatty liver disease in Japanese patients: tight glycemic control, rather than weight reduction, ameliorates liver fibrosis. Diabetes Care.

[27] Do A, Kuszewski EJ, Langberg KA, Mehal WZ (2019). Incorporating weight loss medications into hepatology practice for nonalcoholic steatohepatitis. Hepatology.

[28] Vilar-Gomez E, Martinez-Perez Y, Calzadilla-Bertot L, Torres-Gonzalez A, Gra-Oramas B, Gonzalez-Fabian L (2015). Weight loss through lifestyle modification significantly reduces features of nonalcoholic steatohepatitis. Gastroenterology.

[29] Lassailly G, Caiazzo R, Ntandja-Wandji LC, Gnemmi V, Baud G, Verkindt H (2020). Bariatric surgery provides long-term resolution of nonalcoholic steatohepatitis and regression of fibrosis. Gastroenterology.

[30] Bellentani S, Dalle Grave R, Suppini A, Marchesini G (2008). Fatty liver Italian N. behavior therapy for nonalcoholic fatty liver disease: the need for a multidisciplinary approach. Hepatology.

[31] Short ME, Goetzel RZ, Pei X, Tabrizi MJ, Ozminkowski RJ, Gibson TB (2009). How accurate are self-reports? Analysis of self-reported health care utilization and absence when compared with administrative data. J Occup Environ Med.

[32] Lazarus JV, Palayew A, Carrieri P, Ekstedt M, Marchesini G, Novak K (2021). European 'NAFLD Preparedness Index' - Is Europe ready to meet the challenge of fatty liver disease?. JHEP Rep..

