# Supplementary material for: Nonalcoholic steatohepatitis medical patient journey from the perspective of hepatologists, gastroenterologists and patients: a cross-sectional survey
Source: BMC Gastroenterol. 2022 Jul 10;22:335. doi: 10.1186/s12876-022-02410-x (PMC9272554; doi:10.1186/s12876-022-02410-x)
Supplement: Supplementary file 1 — Additional file 1. NASH_hepgastro_Additional file 1, HCP Survey.pdf HCP Survey, survey used to collect responses from healthcare professionals [file 12876_2022_2410_MOESM1_ESM.pdf]

## Additional file 1: HCP Survey

## SECTION S: SCREENER

### ALL RESPONDENTS

**S0.** Thank you for your interest in this research. We appreciate your willingness to participate in this important research on healthcare issues. Before participating, we require you to review the following information:

- Your responses to this survey will **help the sponsor design new products/services to meet patient needs**.
- Your responses will be kept **strictly confidential** and will never be associated with your name (double-blind).
- We expect, on average, it will take respondents like yourself **25 minutes** to complete this survey.
- Your **participation is voluntary**, and you may choose to stop participating at any time (withdraw consent).

If you qualify for and complete the survey, you will be eligible to receive the honorarium referenced in your invitation.

Do you consent to these terms and wish to continue?

1. Yes **CONTINUE**
2. No **TERMINATE**

[IF CONSENTS TO TERMS (S0r1) ASK AE1. ELSE TERMINATE]

### ALL RESPONDENTS

**AE1.** We are required to pass on to the pharmaceutical company sponsoring the study details of adverse events and/or other safety information - hereinafter referred to as safety information - that are mentioned during this study. Although what you say will be treated in confidence, should you mention safety information during the study, we will need to report it even if you have already reported it to the company or regulatory authorities.

In relation to reporting safety information, situation we need to know if you are willing to waive the confidentiality given to you under the Market Research Codes of Conduct. In the event that you waive confidentiality in relation to safety information reporting, any personal data provided during the reporting will be processed as follows:

a) Any personal data in relation to the safety information reported will be forwarded to the project sponsor; and

b) The project sponsor will record any safety information, including personal data received in the sponsor's global database, in the interests of patient safety and in compliance with all applicable global laws and regulations; and

c) During the reporting of safety information, the project sponsor will not disclose such personal data to any un-associated third parties, with the exception of any disclosures required by applicable law, regulation or the order of a competent authority.

Do you agree to waive the confidentiality given to you under the Market Research Codes of Conduct in relation to any safety information you report to us? If you agree, your contact details will be forwarded to the sponsor's Safety department for the express and sole purpose of follow-up of such report(s). Details of safety information maybe reported to regulatory authorities along with your personal data. All other information provided by you in this study will remain confidential. If you prefer to preserve the confidentiality of this information, please select 'I do not agree'. If you do so, you can still participate in this survey.

1. I agree Please enter your email here: [OPEN TEXT BOX]
2. I do not agree

[IF AGREES (AE1r1) ASK AE2. IF DISAGREES (AE1r2) ASK AE3]

**AGREES ( AE1r1)**
**AE2.** Thank you. Please note that if your email address is provided during the Adverse Event, other safety information or product complaints reporting, this will not be linked in any way to your responses given during the survey.

Are you happy to proceed with this research?

1. Yes **CONTINUE**
2. No **TERMINATE**

[IF YES (AE2/1) CONTINUE TO S3. ELSE TERMINATE]

**DOES NOT AGREE (AE1r2)**
**AE3**. If we become aware of safety information, we are obliged to report this to the pharmaceutical company. We will file this report without giving any of your details.

Are you happy to proceed with this research?

1. Yes **CONTINUE**
2. No **TERMINATE**

[IF HAPPY TO PROCEED (AE3/1) ASK S3. ELSE TERMINATE]

**AGREES TO AE REPORTING (AE2r1 OR AE3r1)**
**S3** To begin, we would like to gather some basic information to be used for categorization purposes.

Which of the following best describes your professional title?

[ALPHA SORT]
1. Physician **CONTINUE**
2. Nurse Practitioner **TERMINATE**
3. Physician Assistant **TERMINATE**
4. Physical Therapist **TERMINATE**

5. Nurse **TERMINATE**
6. Medical Assistant **TERMINATE**
7. Other, please specify [MANDATORY TEXT BOX] [ANCHOR] **TERMINATE**

[IF PHYSICIAN (S3r1) ASK S5. ELSE TERMINATE]

**PHYSICIAN (S3r1)**
**S5.** How many years have you been in practice beyond your residency or fellowship?

*If you are still in your residency, are currently a fellow, or have not been in practice for at least one year, please enter "0" (zero).*

[RANGE: 0-50]

1. |_|_|_| # of years

[IF IN PRACTICE 3-25 YEARS (S5=3-25) ASK S10. ELSE MARK AS NOT QUALIFIED AND CONTINUE]

**PHYSICIAN (S3r1)**
**S10** What is your primary medical specialty?

[ALPHA SORT; GROUP CODES 5,7]

1. Family Practice **CONTINUE**
2. General Practice **CONTINUE**
3. Internal Medicine **CONTINUE**
4. Gastroenterology **CONTINUE**
5. Hepatology **CONTINUE**
6. Endocrinology **CONTINUE**
7. Transplant Hepatology **CONTINUE**
8. Cardiology **TERMINATE**
9. Rheumatology **TERMINATE**
10. Orthopedic **TERMINATE**
11. Pulmonology **TERMINATE**
12. Neurology **TERMINATE**
13. Nephrology **TERMINATE**
14. General Surgery **TERMINATE**
15. Bariatrics Medicine **TERMINATE**
16. Obesity Medicine **TERMINATE**
17. Other, please specify: [MANDATORY TEXT BOX. ANCHOR] **TERMINATE**

[IF PHYSICIAN AND QUALIFIED SPECIALTY (S3/1 AND S10r1-7) ASK.. ELSE MARK AS NOT QUALIFIED AND CONTINUE]

**PHYSICIAN (S3r1)**
**S10A**. Are you board certified or board eligible in your primary specialty?

1. Yes
2. No

[IF BOARD CERTIFIED IN SPECIALTY (S10A/1) CONTINUE. ELSE MARK AS NOT QUALIFIED AND CONTINUE]

**PHYSICIAN (S3r1)**
**S15.** In what state is the facility where you primarily work?

*If you work in more than one state, please select the state where you work the majority of the time.*

[INSERT STATE DROP DOWN]

**PHYSICIAN (S3r1)**
**S20** HIDDEN QUESTION FOR REGION

1. Northeast
   [S15=CT, MA, NH, NJ, NY, PA, RI, VT]
2. Midwest
   [S15=IA, IL, IN, KS, MI, MN, MO, ND, NE, OH, SD, WI]
3. South
   [S15=AL, AR, DC, DE, FL, GA, KY, LA, MD, MS, NC, OK, SC, TN, TX, VA, WV]
4. West
   [S15=AK, AZ, CA, CO, HI, ID, MT, NM, NV, OR, UT, WA, WY]
5. Outside of US
   [ALL OTHERS]

[CONTINUE IF PRACTICES IN THE US BUT NOT VT OR MAINE (S20/1-4 AND S15/NE ME or VT). ELSE MARK AS NOT QUALIFIED AND CONTINUE.]

**PHYSICIAN (S3r1)**
**S30** Which of the following best describes the facility where you primarily see patients?

[ALPHA SORT]

| | |
|---|---|
| 1. Academic hospital (teaching hospital/university) | **CONTINUE** |
| 2. Community hospital | **CONTINUE** |
| 3. Private solo practice | **CONTINUE** |
| 4. Hospital affiliated practice | **CONTINUE** |
| 5. Private single-specialty group practice | **CONTINUE** |
| 6. Private multi-specialty group practice | **CONTINUE** |
| 7. Ambulatory surgery center | **TERMINATE** |
| 8. Government or VA hospital | **TERMINATE** |
| 9. Other, please specify [MANDATORY TEXT BOX] [ANCHOR] | **TERMINATE** |

[CONTINUE IF NOT BASED IN A GOVT/VA HOSPITAL OR ASC OR OTHER FACILITY (S30rNE7-9). ELSE MARK AS NOT QUALIFIED AND CONTINUE]

**PHYSICIAN (S3r1)**
**S31A** In the past month, approximately how many total adult patients (age 18 and older) did you personally see/treat across all conditions and across all care settings (hospitals, outpatient clinics, etc.)?

*Your best estimate is fine.*

[RANGE: 0-9999]

Patient(s) in the past month [_|_I_I_]

**PHYSICIAN (S3r1)**

**S31B** Now, thinking back prior to the COVID-19 pandemic, how many total adult patients (age 18 and older) would you personally see/treat across all conditions and across all care settings (hospitals, outpatient clinics, etc.) in a typical month?

*Your best estimate is fine.*

[RANGE: 0-9999]

Patient(s) seen in typical month [_|_I_I_]

[CONTINUE IF SEES AT LEAST 300 PATIENTS IN TYPICAL MONTH (S31B>299). ELSE MARK AS NOT QUALIFIED AND CONTINUE.]

**PHYSICIAN (S3r1)**

**S35** Considering the [INSERT S31B] adult patients you would see in a typical month prior to the COVID-19 pandemic, approximately how many of these patients are diagnosed with each of the following conditions?

*Please use your best estimate. Your responses may sum to more than [INSERT S31B] to account for patients with multiple conditions. Your responses may sum to less than [INSERT S31B] to account for not all patients having the conditions listed*

[ALPHA-SORT; GROUP CODES 1-2; RANGE: 0-S31B IN EACH CELL;]

1. Non-alcoholic steatohepatitis (NASH) (with or without fibrosis) |_|_|_|
2. Non-alcoholic fatty liver disease (NAFLD) |_|_|_|
3. Type 2 Diabetes |_|_|_|
4. Cardiovascular disease |_|_|_|
5. Hypertension |_|_|_|
6. Dyslipidemia |_|_|_|
7. Pre-diabetes |_|_|_|
8. Cancer |_|_|_|
9. Obesity |_|_|_|

**PHYSICIAN (S3r1)**

**S35A HIDDEN QUESTION – NASH TOTAL PATIENTS**

1. Total NASH patients (SUM S35r1-2)

**PHYSICIAN (S3r1)**

**S36** You indicated that you treat [INSERT S35r1] patients with Non-alcoholic steatohepatitis (NASH) in a typical month. How familiar are you with NASH?

*Please use the scale below where '1' indicates "Not at all familiar", and '7' indicates "Extremely familiar."*

Not at all Extremely

| | | | | | | |
|---|---|---|---|---|---|---|
| familiar | | | | | | familiar |
| 1 | 2 | 3 | 4 | 5 | 6 | 7 |

**PHYSICIAN (S3/1)**
**S40.** Do you or does a member of your immediate family work full or part-time, or consult for any of the following?

*Please select all that apply*.

[MULTIPLE SELECT]

1. Pharmaceutical manufacturer
2. Market research firm
3. Advertising firm
4. None of the above [EXCLUSIVE]

[IF NOT COMPETITIVELY EMPLOYED (S40/4) ASK S100. ELSE MARK AS NOT QUALIFIED AND CONTINUE]

**ALL RESPONDENTS**

**S100 HIDDEN QUOTA QUESTIONS**

1. **QUALIFIED PCP** **N=100**
   - PHYSICIAN (S3r1)
   - IN PRACTICE 3-25 YEARS (S5=3-25)
   - FP/GP/IM (S10r1-3)
   - BOARD CERTIFIED IN SPECIALTY (S10Ar1)
   - NOT BASED IN A GOVT/VA HOSPITAL OR ASC (S30rNE7-9)
   - PRACTICES IN THE US BUT NOT VT OR MAINE (S20r1-4 AND S25rNE ME or VT)
   - SEES AT LEAST 300 PATIENTS IN TYPICAL MONTH (S31B>299)
   - SEES AT LEAST 5 PATIENTS WITH NASH IN TYPICAL MONTH (S35r1>4)
   - FAMILIAR WITH NASH (S36r5-7)
   - NOT COMPETITIVELY EMPLOYED (S40r4)

2. **QUALIFIED ENDOCRINOLOGIST** **N=50**
   - PHYSICIAN (S3r1)
   - IN PRACTICE 3-25 YEARS (S5=3-25)
   - ENDOCRINOLOGY SPECIALTY (S10r6)
   - BOARD CERTIFIED IN SPECIALTY (S10Ar1)
   - NOT BASED IN A GOVT/VA HOSPITAL OR ASC (S30rNE7-9)
   - PRACTICES IN THE US BUT NOT VT OR MAINE (S20r1-4 AND S25rNE ME or VT)
   - SEES AT LEAST 300 PATIENTS IN TYPICAL MONTH (S31A>299)
   - SEES AT LEAST 20 PATIENTS WITH NASH IN TYPICAL MONTH (S35r1>19)
   - FAMILIAR WITH NASH (S36r5-7)
   - NOT COMPETITIVELY EMPLOYED (S40r4)

3. **QUALIFIED GASTROENTEROLOGIST/HEPATOLOGIST** **N=75**
   - PHYSICIAN (S3r1)
   - IN PRACTICE 3-25 YEARS (S5=3-25)
   - GASTROENTEROLOGY OR HEPATOLOGY OR TRANSPLANT HEPATOLOGY SPECIALTY (S10r4-5,7)
   - BOARD CERTIFIED IN SPECIALTY (S10Ar1)
   - NOT BASED IN A GOVT/VA HOSPITAL OR ASC (S30rNE7-9)
   - PRACTICES IN THE US BUT NOT VT OR MAINE (S20r1-4 AND S25rNE ME or VT)
   - SEES AT LEAST 300 PATIENTS IN TYPICAL MONTH (S31A>299)
   - SEES AT LEAST 20 PATIENTS WITH NASH IN TYPICAL MONTH (S35r1>19)
   - FAMILIAR WITH NASH (S36r5-7)
   - NOT COMPETITIVELY EMPLOYED (S40r4)

4. **NOT QUALIFIED**

**QUALIFIED GASTROENTEROLOGIS/HEPATOLOGIST (S100r3)**

**S105 QUOTA GASTRO/HEP SPECIALIST**

1. Gastroenterology (S10r4) N=999
2. Hepatology (S10r5) MAX N=15

3. Transplant Hepatology (10r7) MAX N=10

| SECTION 200: DIAGNOSIS |
| --- |

**ALL QUALIFIED RESPONDENTS (S100r1)**
**Q200** You have qualified for the full survey. Thank you for your responses thus far. The remainder of this survey should take approximately 22 minutes to complete. As a reminder, your responses to this survey are critical to the success of this research in helping the sponsor design new products/services to help you support your patients' needs. Your responses will be kept strictly confidential and only reported in combination with other respondents' data. In addition, you may be asked certain questions for quality control purposes.

**ALL QUALIFIED RESPONDENTS (S100r1)**
**Q205** For the remainder of the survey please focus on your patients who are **diagnosed with Non-alcoholic steatohepatitis (NASH)**.

You indicated you see [INSERT S35r1] adult patients per month who have NASH. Approximately what proportion of these patients fall into each of the age ranges below?

*Your best estimate will do. Your answers must sum to 100%.*

[RANGE 0-100. SHOW CONSTANT SUM INDICATOR. FORCE SUM 100]

| | Proportion of patients with NASH |
| --- | --- |
| 1. Age 18-30 | \|_\|_\|_\|% |
| 2. Age 31-40 | \|_\|_\|_\|% |
| 3. Age 41-50 | \|_\|_\|_\|% |
| 4. Age 51-60 | \|_\|_\|_\|% |
| 5. Over 60 years old | \|_\|_\|_\|% |

**ALL QUALIFIED RESPONDENTS (S100r1)**
**Q207** Next, what proportion of your [INSERT S35r1] adult patients per month who have NASH classify themselves by each of the following gender designations?

*Your best estimate will do. Your answers must sum to 100%.*

[RANGE 0-100. SHOW CONSTANT SUM INDICATOR. FORCE SUM 100]

| | Proportion of patients with NASH |
| --- | --- |
| 1. Male | \|_\|_\|_\|% |
| 2. Female | \|_\|_\|_\|% |
| 3. Other | \|_\|_\|_\|% |
| 4. Not sure [EXCLUSIVE; BUTTON] | |

**ALL QUALIFIED RESPONDENTS (S100r1)**

**Q208** Now, what proportion of your [INSERT S35r1] adult patients per month who have NASH are classified by each of the following ethnicities?

*Your best estimate will do. Your answers must sum to 100%.*

[RANGE 0-100. SHOW CONSTANT SUM INDICATOR. FORCE SUM 100]

| | | Proportion of patients with NASH |
|---|---|---|
| 1. | White | \|_\|_\|_\|% |
| 2. | Black or African American | \|_\|_\|_\|% |
| 3. | Spanish/Hispanic or Latino | \|_\|_\|_\|% |
| 4. | American Indian or Alaska Native | \|_\|_\|_\|% |
| 1. | Asian (i.e., Far East, Southeast Asia, or the Indian subcontinent) | \|_\|_\|_\|% |
| 5. | Other \|_\|_\|_\|% | |
| 6. | Not sure [EXCLUSIVE; BUTTON] | |

**ALL QUALIFIED RESPONDENTS (S100r1)**

**Q209** Approximately what proportion of your [INSERT S35r1] adult patients per month who have NASH also have each of the following comorbidities?

*Your best estimate will do. Your answers do not have to sum to 100%.*

| [ALPHA-SORT] | | Proportion of patients with NASH |
|---|---|---|
| 1. | Hepatitis B or C | \|_\|_\|_\|% |
| 2. | Autoimmune hepatitis | \|_\|_\|_\|% |
| 3. | Sleep apnea | \|_\|_\|_\|% |
| 4. | Anxiety | \|_\|_\|_\|% |
| 5. | Obesity | \|_\|_\|_\|% |
| 6. | Type 1 diabetes | \|_\|_\|_\|% |
| 7. | Type 2 diabetes | \|_\|_\|_\|% |
| 8. | Pre-diabetes | \|_\|_\|_\|% |
| 9. | Cardiovascular / heart disease (including heart failure) | \|_\|_\|_\|% |
| 10. | Depression | \|_\|_\|_\|% |
| 11. | Hypertension (high blood pressure) | \|_\|_\|_\|% |
| 12. | Osteoarthritis (OA) | \|_\|_\|_\|% |
| 13. | Dyslipidemia (high cholesterol) | \|_\|_\|_\|% |
| 14. | Hypertriglyceridemia (high triglycerides) | \|_\|_\|_\|% |
| 15. | Cancer | \|_\|_\|_\|% |
| 16. | PCOS (Polycystic ovary syndrome) and infertility in women | \|_\|_\|_\|% |
| 17. | Other, please specify [ANCHOR] [MANDATORY TEXT BOX] | \|_\|_\|_\|% |
| 18. | None of the above [ANCHOR. EXCLUSIVE] | |

**ALL QUALIFIED RESPONDENTS (S100r1)**
**Q210** What proportion of your [INSERT S35r1] adult patients per month who have NASH have a confirmed diagnosis through biopsy, confirmed diagnosis through other diagnostics, and what proportion have a suspected diagnosis that has not been clinically confirmed?

*Your best estimate will do. Your answers must sum to 100%.*

[RANGE 0-100. SHOW CONSTANT SUM INDICATOR. FORCE SUM 100]

| | | Proportion of patients with NASH |
|---|---|---|
| 1. | Confirmed diagnosis through biopsy | \|_\|_\|_\|% |
| 2. | Confirmed diagnosis through other diagnostics (imaging, bloodwork, etc.) | \|_\|_\|_\|% |
| 3. | Suspected diagnosis not yet clinically confirmed | \|_\|_\|_\|% |

**ALL QUALIFIED RESPONDENTS (S100r1)**
**Q215** Of your [INSERT S35r1] adult patients per month who have NASH, what proportion were diagnosed in the following ways:

*Your best estimate will do. Your answers must sum to 100%.*

[RANGE 0-100. SHOW CONSTANT SUM INDICATOR. FORCE SUM 100]

| | | Proportion of patients with NASH |
|---|---|---|
| 1. | Diagnosed by me | \|_\|_\|_\|% |
| 2. | Referred to me for diagnosis | \|_\|_\|_\|% |
| 3. | Referred out by me for diagnosis | \|_\|_\|_\|% |

**ALL QUALIFIED RESPONDENTS (S100r1)**
**Q220** Finally, of your [INSERT S35r1] adult patients per month who have NASH, what proportion were diagnosed due to their experiencing NASH symptoms, and what proportion were diagnosed for some other reason (i.e., they were asymptomatic)?

*Your best estimate will do. Your answers must sum to 100%.*

[RANGE 0-100. SHOW CONSTANT SUM INDICATOR. FORCE SUM 100]

| | | Proportion of patients with NASH |
|---|---|---|
| 1. | Diagnosed due to experiencing NASH symptoms | \|_\|_\|_\|% |
| 2. | Diagnosed for some other reason (asymptomatic) | \|_\|_\|_\|% |

**ALL QUALIFIED RESPONDENTS (S100r1)**
**Q225** Which of the following clinical treatment guidelines, if any, are you aware of for the diagnosis, treatment, or management of **NASH**?

*Please select all that apply.*

[MULTISELECT. RANDOMIZE]
1. American Association for the Study of Liver Diseases (AASLD)
2. European Association for the Study of the Liver (EASL)

3. European Association for the Study of Diabetes (EASD)
4. European Association for the Study of Obesity (EASO)
96. Other, please specify [MANDATORY TEXT BOX. ANCHOR]
97. I am not aware of any guidelines for NASH [EXCLUSIVE][ANCHOR]

[IF AWARE OF GUIDELINES AND DIAGNOSE AT LEAST SOME NASH PATIENTS (Q225r1-96 ANY AND Q215r3<100) ASK Q226. ELSE SKIP TO Q230]

**AWARE OF GUIDELINES AND DIAGNOSE AT LEAST SOME NASH PATIENTS (Q225r1-96 ANY AND Q215r3<100)**

**Q226** Of the NASH guidelines you are aware of, which do you consider when **diagnosing NASH?**

*Please select all that apply.*

[MULTI-SELECT; SHOW IN SAME ORDER AS Q225; ONLY SHOW THOSE SELECTED AT Q225]

1. American Association for the Study of Liver Diseases (AASLD)
2. European Association for the Study of the Liver (EASL)
3. European Association for the Study of Diabetes (EASD)
4. European Association for the Study of Obesity (EASO)
5. INSERT Q225r96
97. I do not follow any guidelines for the diagnosis of NASH [EXCLUSIVE][ANCHOR]

**DIAGNOSE AT LEAST SOME NASH PATIENTS (Q215r3<100)**

**Q230** When you suspect a patient may have NASH, what tools or clinical tests do you typically use to confirm a diagnosis?

*Please select all that apply.*

[ALPHA-SORT; MULTI-SELECT]

1. Liver function tests (LFTs)
2. Ultrasound
3. MRI-PDPF
4. CT scan
5. FibroScan
6. Liver biopsy
7. Fib-4
8. APRI
9. FibroSure
10. NAFLD/NASH patient screener/self-assessment
11. Other inflammatory markers (APA)
12. A1C levels
13. Lipid levels
14. MRE
96. Other, please specify: [ANCHOR; TEXT BOX]

[IF USES LFTS TO CONFIRM DIAGNOSIS (Q230r1) ASK Q231. ELSE SKIP TO PN AFTER Q231A]

**USES LFTS TO CONFIRM DIAGNOSIS (Q230r1)**
**Q231** When conducting liver function tests (LFTs), how valuable do you find the AST:ALT ratio?

1. Not at all valuable
2. Somewhat valuable
3. Extremely valuable
4. Not sure/Don't know

[IF PROVIDES VALUE RESPONSE (Q231r1-3) ASK Q231A. ELSE SKIP TO PN AFTER Q231A]

**PROVIDES VALUE RESPONSE (Q231r1-3)**
**Q231A** For what reason(s) did you state that you find the AST:ALT ratio to be [INSERT Q231]?

*Please be as detailed as possible.*

[LARGE MANDATORY TEXT BOX]

**DIAGNOSE AT LEAST SOME NASH PATIENTS (Q215r3<100)**
**Q232** When determining a patient's NASH diagnosis, do you make the distinction in classifying it as NASH with fibrosis vs. NASH without fibrosis?

1. Yes
2. No

**MAKE DISTINCTION IN NASH +/- FIBROSIS (Q232r1)**
**Q233** What proportion of your [INSERT S35r1] adult patients per month who have NASH have NASH with fibrosis and what proportion have NASH without fibrosis?

*Your best estimate will do. Your answers must sum to 100%.*

[RANGE 0-100. SHOW CONSTANT SUM INDICATOR. FORCE SUM 100]

| | Proportion of patients with NASH |
|---|---|
| 1. NASH with fibrosis | \|_\|_\|_\|% |
| 2. NASH without fibrosis | \|_\|_\|_\|% |
| 3. Unsure | \|_\|_\|_\|% |

**MAKE DISTINCTION IN NASH +/- FIBROSIS (Q232r1)**
**Q234** How important is the distinction of a patient having NASH with fibrosis vs. without fibrosis when it comes to how you approach and manage NASH?

*Please use a scale below where '1' indicates "Not at all important", and '7' indicates "Extremely important."*

Not at all Extremely

important                                                   important
1 2 3 4 5 6 7

**MAKE DISTINCTION IN NASH +/- FIBROSIS (Q232r1)**

**Q235** How important is the distinction of a patient having NASH with **fibrosis** vs. with **cirrhosis** when it comes to how you approach and manage NASH?

*Please use a scale below where '1' indicates "Not at all important", and '7' indicates "Extremely important."*

| Not at all important | | | | | | Extremely important |
|---|---|---|---|---|---|---|
| 1 | 2 | 3 | 4 | 5 | 6 | 7 |

[IF REFERS PATIENTS OUT FOR DIAGNOSIS (Q215r3>0) ASK Q240. ELSE SKIP TO Q245].

**REFERS PATIENTS OUT FOR DIAGNOSIS (Q215r3>0)**

**Q240** You indicated that you will refer patients out to another physician to diagnose NASH. What providers have you ever referred a patient to in order to confirm a NASH diagnosis?

*Please select all that apply.*

[MULTI-SELECT; ALPHA ORDER]

1. [IF S100r1) "Another"] Primary Care Physician
2. [IF S105r1) "Another"] Gastroenterologist
3. [IF S105r2) "Another"] Hepatologist
4. [IF S100r2) "Another"] Endocrinologist
5. Radiologist
6. Other, please specify: [ANCHOR; TEXT BOX]

**DIAGNOSE AT LEAST SOME NASH PATIENTS (Q215r3<100)**

**Q245** When you diagnose a patient who has NASH, or see them for the first time (after diagnosis), which of the following topics do you typically cover in that conversation?

*Please select all that apply.*

[MULTIPLE SELECT]

1. Cause of NASH
2. Treatments for NASH (e.g., lifestyle modifications, comorbidity management)
3. How NASH is related to or impacts other health conditions
4. Progression to fibrosis
5. Progression to chirrhosis
6. Progression to liver cancer
7. Possibility of liver transplant
8. NASH/fibrosis stage improvement
9. Resolution of NASH
10. Referral to a specialist or other healthcare provider for NASH
96. Other, please specify: [MANDATORY TEXT BOX]
97. I do not typically discuss their NASH with them [EXCLUSIVE]

**DIAGNOSE AT LEAST SOME NASH PATIENTS (Q215r3<100)**

**Q250** When you diagnose a patient who has NASH or see them for the first time (after diagnosis), what, if any, resources do you provide to help your patients' understanding or management of NASH?

*Please select all that apply.*

[MULTIPLE SELECT. RANDOMIZE]

1. Reading material (i.e., pamphlet/brochure, book, magazine) about the condition
2. Website
3. Information on local support group
4. Information on advocacy groups
5. Smartphone app
6. Health System support groups
96. Other, please specify: [MANDATORY TEXT BOX] [ANCHOR]
97. I do not provide any resources [EXCLUSIVE][ANCHOR]

[IF PROVIDED WEBSITE (Q250r2) ASK Q251. ELSE SKIP TO PN AFTER Q251.]

**PROVIDED WEBSITE (Q250r2)**

**Q251** You mentioned that you provide your NASH patients with website(s) to help with their understanding of NASH. What specific websites do you provide?

*Please enter all websites you provide.*

[SHOW 1 ANSER TEXT BOX] ONCE TEXT IS ENTERED SHOW NEXT]

1. MANDATORY TEXT BOX
2. MANDATORY TEXT BOX
3. MANDATORY TEXT BOX

[IF PROVIDED SUPPORT OR ADVOCACY GROUP (Q250r3or4) ASK Q252. ELSE SKIP TO 255.]

**PROVIDED SUPPORT GROUP OR ADVOCACY GROUP (Q250r3or4)**
**Q252** You mentioned that you provide your patients with information on local support group(s) and/or advocacy groups to help with their understanding of NASH. What specific group(s) do you provide?

*Please enter all groups you typically provide.*

[SHOW 1 ANSER TEXT BOX] ONCE TEXT IS ENTERED SHOW NEXT]
1. MANDATORY TEXT BOX
2. MANDATORY TEXT BOX
3. MANDATORY TEXT BOX

## SECTION 300: TREATMENT AND MANAGEMENT

**ALL QUALIFIED RESPONDENTS (S100r1)**
**Q300** In this next section of the survey we would like to understand your treatment and management of patients with NASH.

**AWARE OF GUIDELINES (Q225r1-96 ANY)**
**Q301** To begin, of the NASH guidelines you are aware of, which do you follow for the **treatment and management of NASH?**

*Please select all that apply.*

[MULTI-SELECT; SHOW IN SAME ORDER AS Q225; ONLY SHOW THOSE SELECTED AT Q225]

1. American Association for the Study of Liver Diseases (AASLD)
2. European Association for the Study of the Liver (EASL)
3. European Association for the Study of Diabetes (EASD)
4. European Association for the Study of Obesity (EASO)
5. INSERT Q225r96
97. I do not follow any guidelines for the treatment and management of NASH [EXCLUSIVE][ANCHOR]

**ALL QUALIFIED RESPONDENTS (S100r1)**
**Q305** What type of treatment(s) do you personally prescribe or recommend to patients with **NASH**?

*Please provide your response for both newly diagnosed patients (diagnosed within the past year), as well as those who are on ongoing treatment/management.*

*Please select all that apply.*

[COLUMNS]
1. For patients newly diagnosed with NASH
2. For ongoing NASH treatment and management

[ROWS. RANDOMIZE]
1. Lifestyle changes which focus on exercise (e.g., increasing physical activity)
2. Lifestyle changes which focus on diet (e.g., using a specific diet or diet program (Jenny Craig, Weight Watchers, Elimination diets, Keto, Medifast))
3. Prescription weight loss medication (e.g., Saxenda, Xenical, Qsymia, Contrave)
4. Pioglitazone
5. Obeticholic acid
6. GLP-1 RAs
7. SGLT-2is
8. Metformin
9. Supplements (Vitamin E, milk thistle,etc.)
96. Other treatment for newly diagnosed patients, please specify [MANDATORY TEXT BOX. ANCHOR]
97. Other treatment for ongoing treatment, please specify [MANDATORY TEXT BOX. ANCHOR]
98. I do not prescribe or recommend treatment for NASH [ANCHOR, EXCLUSIVE]

**ALL QUALIFIED RESPONDENTS (S100r1)**
**Q310** How important are each of the following treatment goals to you when managing NASH patients?

*Please use a scale below where '1' indicates "Not at all important", and '7' indicates "Extremely important."*

[COLUMNS]

| Not at all important | | | | | | Extremely important |
|---|---|---|---|---|---|---|
| 1 | 2 | 3 | 4 | 5 | 6 | 7 |

[ROWS; RANDOMIZE]
1. NASH/fibrosis stage improvement
2. Resolution of NASH
3. Delayed NASH progression
4. Delayed progression of fibrosis stage
5. Delayed progression to cirrhosis
6. Prevention of liver transplantation
7. Improvement of comorbidities
8. Weight loss
9. Improved liver function

**ALL QUALIFIED RESPONDENTS (S100r1)**
**Q315** Thinking of your patients who have NASH, approximately what proportion of these patients do you treat personally or refer for treatment of NASH?

*Your best estimate will do.*

[RANGE 0-100. DO NOT FORCE SUM. DO NOT FORCE ZEROS]

| | Proportion of patients with NASH |
|---|---|
| 1. I initiate NASH treatment for patients | \|_\|_\|_\|% |
| 2. I monitor/adjust NASH treatment for patients initiated by someone else | \|_\|_\|_\|% |
| 3. I refer to someone else for treatment of NASH | \|_\|_\|_\|% |

[IF REFERS SOME PATIENTS FOR TREATMENT (Q315r3>0) ASK Q320. ELSE SKIP TO Q330]

**REFERS SOME PATIENTS FOR TREATMENT (Q315r3>0)**
**Q320** What percent of your patients with NASH do you typically refer to each type of healthcare provider listed below for ongoing treatment and management of NASH?

*Your responses do not need to add to 100%.*

| [ALPHA SORT. DO NOT FORCE ZEROS. RANGE 0-100] | Proportion of patients with NASH |
|---|---|
| 1. [IF PCP S100r1 "Another"] Primary Care Provider | \|_\|_\|_\|% |
| 2. [IF S105r1 "Another"] Gastroenterologist | \|_\|_\|_\|% |
| 3. [IF S105r2 "Another"] Hepatologist | \|_\|_\|_\|% |
| 4. [IF S100r2 "Another"] Endocrinologist | \|_\|_\|_\|% |
| 5. Bariatric Surgeon | \|_\|_\|_\|% |
| 6. Obesity Medicine Specialist | \|_\|_\|_\|% |
| 7. Registered Dietitian/Nutritionist | \|_\|_\|_\|% |

96. Other provider |_|_|_|%

[IF REFER TO OTHER PROVIDER (Q320R96>0) ASK Q322. IF REFER TO SPECIALISTS FOR TX/MANAGEMENT (Q320 ANY>0) ASK Q325. ELSE SKIP TO Q330]

**REFER TO OTHER PROVIDER (Q320r96>0)**

**Q322** To what other provider do you refer?

[MANDATORY TEXT BOX]

**HCPS WHO REFER TO SPECIALISTS FOR TX/MANAGEMENT (Q320 ANY>0)**

**Q325** For each specialist that you refer your patients with NASH to for <u>ongoing treatment and management of NASH</u>, what are the primary reason(s) for doing so?

*I primarily refer to this type of provider because the patient…*

[COLUMNS; SHOW ONLY IF RESPONSE AT Q260 >0]

1. PCP
2. Gastroenterologist
3. Hepatologist
4. Endocrinologist
5. Bariatric Surgeon
6. Obesity Medicine
7. Dietitian/Nutritionist
8. [INSERT Q322 IF Q320R96>0]

[ROWS, MULTISELECT.]

1. [DO NOT USE]
2. Additional management/treatment of NASH
3. Needs to lose weight (i.e., to undergo surgery, to improve NASH prognosis and progression)
4. Struggles with lifestyle modifications
5. Needs treatment for comorbidities (e.g., type 2 diabetes, cardiovascular disease, obesity)
6. Needs treatment for other NASH symptoms outside of my specialty
7. The patient's NASH symptoms have worsened
8. Worsening of NASH, regardless of symptoms

96. Other reason [POP UP TEXT BOX ON NEXT PAGE IF SELECTED]

**HCPS WHO REFER TO SPECIALISTS FOR TX/MANAGEMENT (Q320 ANY>0)**

**Q326** For what reasons do your patients typically believe is the reason they are being referred to a specialist?

*Please select all that apply.*

[MULTI-SELECT; SHOW IN SAME ORDER AS Q325]

1. [DO NOT USE]
2. Additional management/treatment of NASH
3. Needs to lose weight (i.e., to undergo surgery, to improve NASH prognosis and progression)
4. Struggles with lifestyle modifications
5. Needs treatment for comorbidities (e.g., type 2 diabetes, cardiovascular disease, obesity)
6. Needs treatment for other NASH symptoms outside of my specialty
7. The patient's NASH symptoms have worsened

8. Worsening of NASH, regardless of symptoms
97. Other reason [POP UP TEXT BOX ON NEXT PAGE IF SELECTED]

**ALL QUALIFIED RESPONDENTS (S100r1)**
**Q330** What proportion of your NASH patients, if any, were referred to you specifically <u>for treatment</u>?

| | Proportion of patients with NASH |
|---|---|
| 1. Patients referred to me for treatment | |_|_|_|% [RANGE 0-100] |

[IF PATIENTS REFERRED (Q330>0) ASK Q335. ELSE SKIP TO Q340.]

**PATIENTS REFERRED (Q330>0)**
**Q335** You indicated that patients are referred to you for NASH treatment. What proportion of these patients are referred from each of the following healthcare providers?

*Your responses must add to 100%.*

[ALPHA SORT. DO NOT FORCE ZEROS. RANGE 0-100;SHOW CONSTANT SUM INDICATOR]

| | Proportion of patients with NASH |
|---|---|
| 1. [IF PCP S100r1 "Another"] Primary Care Provider | |_|_|_|% |
| 2. [IF S105r1 "Another"] Gastroenterologist | |_|_|_|% |
| 3. [IF S105r2 "Another"] Hepatologist | |_|_|_|% |
| 4. [IF S100r2 "Another"] Endocrinologist | |_|_|_|% |
| 5. Bariatric Surgeon | |_|_|_|% |
| 6. Obesity Medicine Specialist | |_|_|_|% |
| 7. Registered Dietitian/Nutritionist | |_|_|_|% |
| 97. Other provider | |_|_|_|% |

**ALL QUALIFIED RESPONDENTS (S100r1)**
**Q340** What type of healthcare provider do you typically think of as the "coordinator of care" of patients who have NASH?

*By "coordinator" please consider the healthcare provider who is primarily responsible for managing the patient's NASH in conjunction with any other comorbidities they have.*

[ROWS, ALPHA SORT.]

1. I am the coordinator of care [ANCHOR AT TOP]
2. [IF PCP S100r1 "Another"] Primary Care Provider
3. [IF S105r1 "Another"] Gastroenterologist
4. [IF S105r2 "Another"] Hepatologist
5. [IF S100r2 "Another"] Endocrinologist
6. Bariatric Surgeon
7. Obesity Medicine Specialist
8. Registered Dietitian/Nutritionist
9. Other provider; please specify [ANCHOR; TEXT BOX]

**ALL QUALIFIED RESPONDENTS (S100r1)**
**Q345** In your opinion, what are the **top three** reasons patients who have NASH **discontinue the treatment and management of their NASH**?

*Please select up to three responses.*

[RANDOMIZE. MULTIPLE SELECT. ONLY ALLOW THREE SELECTIONS]
1. Personal financial limitations
2. Change in insurance coverage
3. Lack of treatment efficacy
4. Unwilling to comply with recommended lifestyle changes
5. Transportation difficulties
6. Expense of multiple visits
7. Improvement in symptoms
8. Difficulty with lifestyle recommendation compliance
9. No accountability
10. Asymptomatic condition – out of sight, out of mind
11. I do not ask/require them to schedule a follow-up
96. Other, please specify [INSERT TEXT BOX. ANCHOR]

**ALL QUALIFIED RESPONDENTS (S100r1)**
**Q350** To what extent to do you feel a patient's comorbidities impact each of the following regarding NASH?

*Please use the below scale where "1" means "Doesn't impact at all", and 7 means "Greatly impacts."*

[COLUMNS]

| Doesn't impact At all | | | | | | Greattly impacts |
|---|---|---|---|---|---|---|
| 1 | 2 | 3 | 4 | 5 | 6 | 7 |

[ROWS; RANDOMIZE]
1. Initial development of NASH
2. Progression of NASH
3. Regression of NASH
4. NASH Prognosis

## SECTION 400: NASH PROGRESSION

**ALL QUALIFIED RESPONDENTS (S100r1)**
**Q401** How frequently do you typically have follow-up appointments specific to NASH with your patients after diagnosis?

1. Weekly
2. Monthly
3. Bi-monthly (every other month)
4. Quarterly (every 3 months)
5. Bi-annually (every 6 months)
6. Yearly

96. Other, please specify: [MANDATORY TEXT BOX. ANCHOR]
97. I typically do not have follow-up appointments with patients specific to their NASH [EXCLUSIVE] [ANCHOR]

**ALL QUALIFIED RESPONDENTS (S100r1)**
**Q405** What tools or clinical tests do you typically use to **monitor NASH progression**?

*Please select all that apply.*

[ALPHA-SORT; MULTI-SELECT; GROUP R1-R2]

1. Liver function tests (LFTs)
2. AST:ALT ratio
3. Ultrasound
4. MRI-PDPF
5. CT scan
6. FibroScan
7. Liver biopsy
8. Fib-4
9. APRI
10. FibroSure
11. NAFLD/NASH patient screener/self-assessment
12. Other inflammatory markers (APA)
13. A1C levels
14. Lipid levels
15. MRE
16. [INSERT Q230r96]

96. Other, please specify: [ANCHOR; TEXT BOX]
97. I do not assess NASH progression by clinical means [ANCHOR; EXCLUSIVE]

**THOSE ASSESSING PROGRESSION OF NASH (Q405NE97)**
**Q408** How frequently are you typically monitoring NASH progression in your patients using the following tools or clinical measures?

[COLUMNS]
1. Weekly
2. Monthly
3. Bi-monthly (every other month)
4. Quarterly (every 3 months)
5. Bi-annually (every 6 months)
6. Yearly
7. [INSERT Q401r96]

[ROWS]
[ALPHA-SORT; SHOW THOSE SELECTED IN Q405; GROUP R1-R2]
1. Liver function tests (LFTs)
2. AST:ALT ratio
3. Ultrasound
4. MRI-PDPF
5. CT scan
6. FibroScan
7. Liver biopsy
8. Fib-4
9. APRI
10. FibroSure
11. NAFLD/NASH patient screener/self-assessment
12. Other inflammatory markers (APA)
13. A1C levels
14. Lipid levels
15. [INSERT Q230r96]
96. Other, please specify: [ANCHOR; TEXT BOX]

## SECTION 500: PATIENT RELATIONSHIPS

**ALL QUALIFIED RESPONDENTS (S100r1)**
**Q500** Switching gears, we would like to understand the relationships you have with your NASH patients.

To begin, which of the following would you use to describe your "style" or role in managing patients with NASH?

*Please select all that apply.*

[RANDOMIZE; MULTI-SELECT]
1. Calm
2. Approachable
3. Informative
4. Partner
5. Listener
6. Advocate
7. Blunt
8. Strict
9. Direct
10. Honest
11. Encouraging
12. Proactive
13. Other, please specify: [ANCHOR; TEXT BOX]

**ALL QUALIFIED RESPONDENTS (S100r1)**
**Q502** Thinking about your NASH patients, to what extent do you agree that the relationship between you and a particular patient impacts their success in managing NASH.

*Please use the below scale where "1" means "Don't agree at all", and 7 means "Completely agree."*

| Don't agree At all | | | | | | Completely agree |
|---|---|---|---|---|---|---|
| 1 | 2 | 3 | 4 | 5 | 6 | 7 |

**ALL QUALIFIED RESPONDENTS (S100r1)**
**Q505** Over time, what impact, if any, do each of the following have on the relationship that you have with your patients with NASH?

*Please indicate the degree to which each of the items below have a positive, negative, or no impact on the doctor-patient relationship.*

[COLUMNS]

| Negatively Impacts relationship | | | Doesn't impact relationship | | | Positively impacts relationship |
|---|---|---|---|---|---|---|
| 1 | 2 | 3 | 4 | 5 | 6 | 7 |

[ROWS]
1. Patient has success with NASH treatment
2. Patient has challenges with NASH treatment
3. Patient is compliant with treatment
4. I gain a better understanding of their comborbidities
5. High level of patient motivation to improve NASH
6. Low level of patient motivation to improve NASH

**ALL QUALIFIED RESPONDENTS (S100r1)**
**Q545** What are the **top 5 types of support that would be most helpful for your patients with NASH** to be successful in managing their NASH and any comorbidities?

*Please select your top 5.*

[SELECT 5 ONLY]

[RANDOMIZE]
1. Resources for family and friends to help understand how to be supportive
2. Resources for lifestyle management
3. Support groups (online or in-person)
4. Advocacy groups
5. Programs offered at workplaces
6. Financial support for healthy choices
7. Wellness apps
8. Weekly follow-up visits with a healthcare provider
9. Access to a physician who specializes in NASH
96. Other, please specify: [INSERT TEXT BOX. ANCHOR]
97. Patients don't need any type of support [EXCLUSIVE, ANCHOR]

## SECTION 600: IMPACT OF NASH AND FUTURE TRENDS

**ALL QUALIFIED RESPONDENTS (S100r1)**
**Q600 Now we would like to understand how NASH impacts your patients' life.** How much would you say NASH (and any associated symptoms or requirements for managing it) impacts each of the following aspects of their daily life?

*Please use a scale from 1 to 7 where "1" means it "Doesn't impact at all" and "7" means "Greatly impacts."*

| [RANDOMIZE ROWS] | 1 – Doesn't impact at all | 2 | 3 | 4 | 5 | 6 | 7 – Greatly impacts |
|---|---|---|---|---|---|---|---|
| 1. Social relationships and activities | | | | | | | |
| 2. Family relationships and activities | | | | | | | |
| 3. Finances | | | | | | | |
| 4. Doing household chores (cooking, cleaning, etc.) | | | | | | | |
| 5. Running errands (go to the grocery store, doctors' appointments, etc.) | | | | | | | |
| 6. Job or career | | | | | | | |
| 7. Being active / exercising | | | | | | | |
| 8. Hobbies | | | | | | | |
| 9. Mental / emotional health | | | | | | | |

**ALL QUALIFIED RESPONDENTS (S100r1)**
**Q605** With approximately how many of your NASH patients have you had discussions with in the past about participation in **clinical trials**? How many, if any, have participated?

[COLUMNS]
1. Have discussed
2. Have participated

[ROWS; RANDOMIZE]
1. NASH with fibrosis |_|_|_| [RANGE: 0-999]
2. NASH without fibrosis |_|_|_| [RANGE: 0-999]

**HAVE DISCUSSED NASH CLINICAL TRIALS (Q605c1>0)**
**Q608** What questions, if any, do patients tend to have about clinical trial participation?

*Please select all that apply.*
[RANDOMIZE. MULTI-SELECT]
1. Am I a good candidate
2. How long will the trial last
3. How will I know I'm getting treatment vs. a placebo
4. How much will it cost
5. Where is the trial being held / Where do I have to go
6. Will I be paid for participation
7. Will my other doctors/healthcare providers know that I'm participating
8. What are the potential side effects
9. How will I know if it is working
96. Other, please specify: [INSERT TEXT BOX. ANCHOR]

**ALL QUALIFIED RESPONDENTS (S100r1)**
**Q610** Thinking ahead to the next 5 years, what treatments are you aware of that are in development for the treatment of NASH?

*Please select all that apply.*

[RANDOMIZE; MULTI-SELECT]
1. Resmetirom / MGL-3196 (from Madrigal) THR β-selective agonist
2. Obeticholic acid (from Intercept)
3. efruxifermin (EFX) / AKR-001 (from Akero) FC-FGF21 analog
4. Cenicriviroc (from Allergan) CCR type 2/5 dual antagonist
5. Pegbelfermin (from Bristol Myers Squibb) PEGylated FGF21 analog
6. Elafibranor (from GENFIT) dual agonist of the PPARα and PPARδ
7. Belapectin / GR-MD-02 (from Galectin Therapeutics) galectin-3 inhibitor
8. Aramchol (from Galmed Pharmaceuticals) anti-fibrotic
9. Ozempic (from Novo Nordisk) semaglutide
96. Other, please specify: [ANCHOR; TEXT BOX]
99. I am not aware of any NASH treatments in development [ANCHOR; EXCLUSIVE]

[IF AWARE OF TREATMENTS (Q610rNE99) ASK Q611. ELSE SKIP TO Q615.]

**AWARE OF TREATMENTS (Q610rNE99)**
**Q611** For the treatment(s) you are aware of in development for the treatment of NASH, how promising do you find the following to be as a potential treatment for NASH?

*Please use the below scale where "1" means "Not at all promising", and 7 means "Extremely promising."*

[COLUMNS]

| Not at all promising | | | | | | Extremely promising |
|---|---|---|---|---|---|---|
| 1 | 2 | 3 | 4 | 5 | 6 | 7 |

[ROWS; ONLY SHOW THOSE SELECTED AT Q610; SHOW IN SAME ORDER AS Q610]
1. Resmetirom / MGL-3196 (from Madrigal) THR β-selective agonist
2. Obeticholic acid (from Intercept)
3. efruxifermin (EFX) / AKR-001 (from Akero) FC-FGF21 analog
4. Cenicriviroc (from Allergan) CCR type 2/5 dual antagonist
5. Pegbelfermin (from Bristol Myers Squibb) PEGylated FGF21 analog
6. Elafibranor (from GENFIT) dual agonist of the PPARα and PPARδ
7. Belapectin / GR-MD-02 (from Galectin Therapeutics) galectin-3 inhibitor
8. Aramchol (from Galmed Pharmaceuticals) anti-fibrotic
9. Ozempic (from Novo Nordisk) semaglutide
10. [INSERT Q610r96]

**ALL QUALIFIED RESPONDENTS (S100r1)**
**Q615** Now, imagine there is pharmaceutical treatment for NASH going into clinical trials. Please indicate how interested you would be in referring your patients with NASH to participate in a clinical trial for this treatment if you had the opportunity.

*Use a scale where a "1" indicates "Not at all interested," and a "7" indicates "Extremely interested."*

| Not at all interested | | | | | | Extremely interested |
|---|---|---|---|---|---|---|
| 1 | 2 | 3 | 4 | 5 | 6 | 7 |

**ALL QUALIFIED RESPONDENTS (S100r1)**
**Q616** Still thinking of a pharmaceutical treatment for NASH going into clinical trials, which patients would you consider to be optimal candidates for a clinical trial? NASH patients with. . .

*Please select all that apply.*
[MULTI-SELECT]
1. No scarring (F0)
2. Mild fibrosis (F1)
3. Moderate fibrosis (F2)
4. Severe fibrosis (F3)
5. Cirrhosis (F4)
6. Don't know/Not sure

**ALL QUALIFIED RESPONDENTS (S100r1)**

**Q617** If the following were requirements of your patients as part of participating in a clinical trial for a pharmaceutical treatment for NASH, how willing would your patients be to participate?

*Use a scale where a "1" indicates "Not at all willing," and a "7" indicates "Extremely willing."*

[COLUMNS]

| Not at all willing | | | | | | Extremely willing |
|---|---|---|---|---|---|---|
| 1 | 2 | 3 | 4 | 5 | 6 | 7 |

[ROWS; RANDOMIZE]

1. Liver biopsy
2. Ultrasound
3. CT scan
4. FibroScan
5. Liver Function Tests

**ALL QUALIFIED RESPONDENTS (S100r1)**
**Q620** Still thinking of a pharmaceutical NASH treatment, please indicate how **meaningful** each of the following clinical endpoints would be to you in a clinical trial.

*Use a scale where a "1" indicates "Not at all meaningful," and a "7" indicates "Extremely meaningful."*

| Not at all meaningful | | | | | | Extremely meaningful |
|---|---|---|---|---|---|---|
| 1 | 2 | 3 | 4 | 5 | 6 | 7 |

[ROWS; RANDOMIZE; GROUP R9-11 IN ORDER]
1. Positive Impact on Quality of Life (IQOL)
2. Improvement in NASH symptoms (e.g., less fatigue, reduced abdominal pain, reduction in swelling)
3. NASH/fibrosis stage improvement
4. Delayed progression to cirrhosis
5. Delayed progression of fibrosis stage
6. Decreased A1C levels
7. Improved LFTs
8. 5-10% weight loss
9. 11-15% weight loss
10. 16-20% weight loss
11. Patient tolerability
12. Regression of fibrosis

[SHOW Q630 AND Q631 ON SAME SCREEN]

**ALL QUALIFIED RESPONDENTS (S100r1)**
**Q630** In your view, what would the ideal mode of administration with which patients had to take a new pharmaceutical treatment for NASH be?

1. Injectable (medication that is injected into the muscle (intramuscular) or just under the skin (subcutaneous))
2. Pill
3. Infusion (medication is delivered through a needle placed directly into a vein

**ALL QUALIFIED RESPONDENTS (S100r1)**
**Q631** And what frequency would be most ideal?

1. Daily
2. Weekly
3. Monthly

**ALL QUALIFIED RESPONDENTS (S100r1)**
**Q640** What are your expectations on how **NASH management** will be handled in the next 5 years?

[MANDATORY LARGE TEXT BOX]

## SECTION 100: DEMOGRAPHICS

Just a few more questions for classification purposes.

**ALL QUALIFIED RESPONDENTS (S100r1)**
**Q100** How do you describe yourself?

1. Male
2. Female
3. Transgender
4. Do not identify as female, male or transgender

**ALL QUALIFIED RESPONDENTS (S100r1)**
**Q101** In what year were you born?

*Please enter as a four-digit number, e.g., 1963.*

[RANGE: 1890-2015]
|_|_|_|_|

**ALL QUALIFIED RESPONDENTS (S100r1)**
**Q102**
HIDDEN COMPUTE FOR AGE

**ALL QUALIFIED RESPONDENTS (S100r1)**
**Q103** Are you Spanish/Hispanic or Latino?

1. Yes
2. No
3. Decline to answer

**ALL QUALIFIED RESPONDENTS (S100r1)**
**Q104** Are you…?

*Please select all that apply.*

[MULTIPLE SELECT]

1. White
2. Black or African American
3. American Indian or Alaska Native
4. Asian
5. Other, please specify: ________
6. Decline to answer [EXCLUSIVE]

**ALL QUALIFIED RESPONDENTS (S100r1)**
**Q105** Which description best categorizes the setting where you work <u>most often</u>?

1. Urban
2. Suburban
3. Rural

**ALL QUALIFIED RESPONDENTS (S100r1)**
**Q106** Thinking about where you work <u>most often</u>, what percentage of the below insurance coverage makes up your practice?

*Your total must sum to 100%. Please check the box below if you are not sure.*

[RANGE: 0-100; SUM TO 100; CONSTANT SUM; RECODE BLANK TO ZERO]

1. Medicare [_] %
2. Medicaid [_] %
3. Commercial insurance/Private pay [_] %
4. Self-pay/Cash [_] %
5. Other [_] %
6. Not sure [EXCLUSIVE]

**ALL QUALIFIED RESPONDENTS (S100/r-4)**
**Q107** What proportion of your revenue is based on the following types of reimbursement?

*Your total must sum to 100%. Please check the box below if you are not sure.*

[RANGE: 0-100; SUM TO 100; CONSTANT SUM; RECODE BLANK TO ZERO]

1. Value-based [_] %
2. Fee-for-service [_] %
3. Other [_] %
4. Not sure [EXCLUSIVE]
