# Supplementary material for: Nonalcoholic steatohepatitis medical patient journey from the perspective of hepatologists, gastroenterologists and patients: a cross-sectional survey
Source: BMC Gastroenterol. 2022 Jul 10;22:335. doi: 10.1186/s12876-022-02410-x (PMC9272554; doi:10.1186/s12876-022-02410-x)
Supplement: Supplementary file 2 — Additional file 2. NASH_hepgastro_Additional file 2, Patient Survey.pdf Patient Survey, survey used to collect responses from patients with NASH [file 12876_2022_2410_MOESM2_ESM.pdf]

Additional file 2: Patient Survey

SECTION S: SCREENER

ALL RESPONDENTS
S0. Thank you for your interest in this research. We appreciate your willingness to participate in this important research on healthcare issues. Before participating, we require you to review the following information:

- Your responses to this survey will **help the sponsor design new products/services to meet patient needs**.
- Your responses will be kept **strictly confidential** and will never be associated with your name (double-blind).
- We expect, on average, it will take respondents like yourself **25 minutes** to complete this survey.
- Your **participation is voluntary**, and you may choose to stop participating at any time (withdraw consent).

If you qualify for and complete the survey, you will be eligible to receive the honorarium referenced in your invitation.

Do you consent to these terms and wish to continue?

1. Yes **CONTINUE**
2. No **TERMINATE**

[IF CONSENTS TO TERMS (S0r1) ASK AE1. ELSE TERMINATE]

ALL RESPONDENTS
AE1. We are required to pass on to the pharmaceutical company sponsoring the study details of adverse events and/or other safety information - hereinafter referred to as safety information - that are mentioned during this study. Although what you say will be treated in confidence, should you mention safety information during the study, we will need to report it even if you have already reported it to the company or regulatory authorities.

In relation to reporting safety information, situation we need to know if you are willing to waive the confidentiality given to you under the Market Research Codes of Conduct. In the event that you waive confidentiality in relation to safety information reporting, any personal data provided during the reporting will be processed as follows:

a) Any personal data in relation to the safety information reported will be forwarded to the project sponsor; and

b) The project sponsor will record any safety information, including personal data received in the sponsor's global database, in the interests of patient safety and in compliance with all applicable global laws and regulations; and

c) During the reporting of safety information, the project sponsor will not disclose such personal data to any un-associated third parties, with the exception of any disclosures required by applicable law, regulation or the order of a competent authority.

Do you agree to waive the confidentiality given to you under the Market Research Codes of Conduct in relation to any safety information you report to us? If you agree, your contact details will be forwarded to the sponsor's Safety department for the express and sole purpose of follow-up of such report(s). Details of safety information may be reported to regulatory authorities along with your personal data. All other information provided by you in this study will

remain confidential. If you prefer to preserve the confidentiality of this information, please select 'I do not agree'. If you do so, you can still participate in this survey.

1. I agree Please enter your email here: [OPEN TEXT BOX]
2. I do not agree

[IF AGREES (AE1r1) READ AE2. IF DISAGREES (AE2r2) ASK AE3]

AGREES (AE1r1)
AE2. Thank you. Please note that in providing your email address during the Adverse Event, other safety information or product complaints reporting, this will not be linked in any way to your responses given during the survey.

Are you happy to proceed with this research?

1. Yes **CONTINUE**
2. No **TERMINATE**

[IF YES (AE2r1) JUMP TO S1; ELSE TERMINATE]

DOES NOT AGREE (AE1r2)
AE3 If we become aware of safety information, we are obliged to report this to the pharmaceutical company. We will file this report without giving any of your details.

Are you happy to proceed with this research?

1. Yes **CONTINUE**
2. No **TERMINATE**

[IF HAPPY TO PROCEED (AE3r1) ASK S3. ELSE TERMINATE]

AGREES TO REPORTING (AE2r1 OR AE3r1)
S3. Thank you. This research is focused on your personal experiences, and we very much appreciate your honesty in responding. To begin, we would like to gather some basic information to be used for categorization purposes.

In what year were you born?

Please enter as a four-digit number, e.g., 1963.

[RANGE: 1890-2015]
|_|_|_|_|

AGREES TO REPORTING (AE2r1 OR AE3r1)

S4 HIDDEN COMPUTE FOR AGE

[IF AGE 18 OR OLDER (S4>17) ASK S5. ELSE, TERMINATE]

AGE 18 OR OLDER (S4>17)
S5. How do you describe yourself?

1. Male
2. Female
3. Transgender
4. Do not identify as female, male or transgender

AGE 18 OR OLDER (S4>17)
S10. In what state is your residence?

If you have residences in more than one state, please select the state where you spend the majority of the time.

[INSERT STATE DROP DOWN]

LIVES IN THE U.S. (S10/NE99)
S11 HIDDEN QUESTION FOR REGION

1. Northeast
   [S11=CT, MA, ME, NH, NJ, NY, PA, RI, VT]
2. Midwest
   [S11=IA, IL, IN, KS, MI, MN, MO, ND, NE, OH, SD, WI]
3. South
   [S11=AL, AR, DC, DE, FL, GA, KY, LA, MD, MS, NC, OK, SC, TN, TX, VA, WV]
4. West
   [S11=AK, AZ, CA, CO, HI, ID, MT, NM, NV, OR, UT, WA, WY]
5. Outside of US [ALL OTHERS]

[IF RESIDES IN THE US (S11rNE5) ASK S14. ELSE TERMINATE]

RESIDES IN THE US (S11/NE5)
S14. With which, if any, of the following conditions have you ever been diagnosed by a healthcare professional (physician, nurse practitioner, etc.)?

Please select all that apply.

[ALPHA SORT. MULTIPLE SELECT]

1. Non-alcoholic steatohepatitis (NASH)
2. Non-alcoholic fatty liver disease (NAFLD) / Fatty Liver Disease
3. Hepatitis B or C
4. Autoimmune hepatitis
5. Sleep apnea
6. Anxiety
7. Obesity
8. Type 1 diabetes
9. Type 2 diabetes
10. Pre-diabetes (which is a slightly elevated blood glucose levels, regarded as indicative that a person is at risk of progressing to Type 2 diabetes)
11. Cardiovascular / heart disease (including heart failure)
12. Depression
13. Hypertension (high blood pressure)
14. Osteoarthritis (OA)
15. Dyslipidemia (high cholesterol)
16. Chronic obstructive pulmonary disease (COPD)
17. Hypertriglyceridemia (high triglycerides)
18. Cancer
19. PCOS (Polycystic ovary syndrome) and infertility in women
96. Other, please specify [ANCHOR] [MANDATORY TEXT BOX]
97. None of the above [ANCHOR. EXCLUSIVE]

[IF DIAGNOSED WITH NASH (S14R1) ASK S17, ELSE TERMINATE]

DIAGNOSED WITH NASH (S14R1)
S17. You mentioned being diagnosed with non-alcoholic steatohepatitis (NASH). Approximately how long ago were you diagnosed?

Please use your best estimate. If you were diagnosed less than one year ago, please enter zero '0'.

[RANGE: 0-S4]

1. |_|_|_| years since NASH diagnosis

[IF DIAGNOSED WITH NASH WITHIN PAST 10 YEARS (S17<11) CONTINUE. ELSE MARK AS NOT QUALIFIED AND CONTINUE]

DIAGNOSED WITH NASH (S14R1)

S20. What tests or procedures have you undergone to diagnose or monitor your non-alcoholic steatohepatitis (NASH)?

Please select all that apply.

[RANDOMIZE, MULTIPLE SELECT]
1. Liver function tests – *a group of blood tests intended to measure the presence of various liver enzymes*
2. Imaging – *potentially an MRI, MRE, VCTE, CT, or ultrasound, used to visualize organs inside the body (particularly your liver)*
3. FibroScan – *a specific form of medical imaging used to assess the 'hardness' or 'stiffness' of the liver*
4. Liver biopsy – *procedure in which a small needle is inserted into the liver to collect a tissue sample*
5. NASH biomarker panel
6. Other lab test, please specify [MANDATORY TEXT BOX, ANCHOR]
7. Not sure/Don't recall [EXCLUSIVE, ANCHOR]

[IF KNOWS TEST/PROCEDURES DONE (S20rNE7, ASK S27. ELSE MARK AS NOT QUALIFIED AND CONTINUE]

DIAGNOSED WITH NASH (S14R1)
S27. To the best of your knowledge, what level of liver scarring have you been diagnosed with as a result of your non-alcoholic steatohepatitis (NASH)?

1. No scarring (F0)
2. Mild fibrosis (F1)
3. Moderate fibrosis (F2)
4. Severe fibrosis (F3)
5. Cirrhosis (F4)
6. Don't know/Not sure

DIAGNOSED WITH NASH (S14R1)
S28. What type(s) of healthcare provider(s) are you currently seeing for management of your non-alcoholic steatohepatitis (NASH)?

Please select all that apply.

[ALPHA SORT, MULTIPLE SELECT]
1. Primary care physician (PCP) / General Practitioner (GP) / Family Physician (FP) / Internal Medicine (IM)
2. Hepatologist
3. Gastroenterologist
4. Endocrinologist
5. Obstetrician / Gynecologist (OB/GYN)
6. Cardiologist
7. Other, please specify [MANDATORY TEXT BOX. ANCHOR]
8. I am not currently seeing a healthcare provider for my non-alcoholic steatohepatitis (NASH) [EXCLUSIVE. ANCHOR]

[IF BEING TREATED BY AN HCP (S28/NE11). ASK S30. ELSE MARK AS NOT QUALIFIED AND CONTINUE]

DIAGNOSED WITH NASH (S14R1)

Just a few more questions for classification purposes.

S29 Are you Spanish/Hispanic or Latino?

1. Yes
2. No
3. Decline to answer

DIAGNOSED WITH NASH (S14R1)
S30 Are you…?

Please select all that apply.

[MULTIPLE SELECT]
1. White
2. Black or African American
3. American Indian or Alaska Native
4. Asian
5. Other, please specify: ________
6. Decline to answer [EXCLUSIVE]

DIAGNOSED WITH NASH (S14R1)
S31 What is your height (feet, inches)?

Your best estimate will do.

[RANGE 1-9]
1. Feet: |_| and

[RANGE 0-11]
2. Inches: |_|_|

DIAGNOSED WITH NASH (S14R1)
S32 What is your current weight (pounds)?

Please be as exact as possible.

[RANGE 50-1000]
1. Pounds |_|_|_|_|

DIAGNOSED WITH NASH (S14R1)
S33 HIDDEN QUESTION FOR BMI CALCULATION

[BMI = (S31r1*703) / (S32r1*12 + S31r2) ^2]

DIAGNOSED WITH NASH (S14R1)

S35. Do you or does a member of your immediate family work full or part-time, or consult for any of the following?

[MULTIPLE SELECT]

1. Pharmaceutical manufacturer
2. Market research firm
3. Advertising firm
4. None of the above [EXCLUSIVE]

[IF NOT COMPETITIVELY EMPLOYED (S35r4) ASK S100. ELSE TERMINATE]

FINAL QUOTA QUESTIONS

S100 **TOTAL N =300**

1. QUALIFIED NASH PATIENT N = 300
    a. AGE 18+ (S4>17)
    b. RESIDES IN THE US (S11rNE5)
    c. DIAGNOSED WITH NASH (S14r1)
    d. DIAGNOSED WITH NASH WITHIN PAST 10 YEARS (S17<11)
    e. KNOWS TEST/PROCEDURES DONE (S20/NE7)
    f. BEING TREATED BY AN HCP (S28/NE11)
    g. NOT COMPETITIVELY EMPLOYED (S35/4)

2. NOT QUALIFIED N=9999

TIME SINCE DIAGNOSIS QUOTA QUESTION

S105

1. DIAGNOSED LESS THAN 1 YEAR AGO [S17=0]	N=MIN 50
2. DIAGNOSED 1-2 YEARS AGO [S17r1-2]	N=MIN 50
3. DIAGNOSED 3-4 YEARS AGO [S17r3-4]	N=MIN 50
4. DIAGNOSED 5-10YEARS AGO [S17r5-10]	N=MIN 30

METHOD OF DIAGNOSIS <u>SOFT</u> QUOTA QUESTION

S115

1. BIOPSY (S20r4)	N=100
2. OTHER METHOD (S20rNE4)	N=200

RACE/ETHNICITY <u>SOFT</u> QUOTA QUESTION

S120 – LEAST FILL

1. HISPANIC (S29r1)	N=MIN 75
2. CAUCASIAN (S30r1)	N=MIN 50
3. AFRICAN AMERICAN (S30r2)	N=MIN 75
4. ASIAN (S30r4)	N=MIN 50
5. OTHER (S30r3,5,6)	N=999

GENDER <u>SOFT</u> QUOTA QUESTION

S125 GENDER

1. MALE (S5r1)	N=150
2. FEMALE (S5r2)	N=150
3. OTHER (S5NEr1-2)	N=999

AGE <u>SOFT</u> QUOTA QUESTION

S130 AGE

1. AGE 50 OR YOUNGER (S4r18-50)	N=100
2. AGE 51-60 (S4r51-60)	N=100
3. AGE 61+ (S4>60)	N=100

SECTION 200: PRE-DIAGNOSIS

ALL QUALIFIED RESPONDENTS (S100r1)
Q200 Congratulations! You have qualified for the full survey. Thank you for your responses thus far. The remainder of this survey should take approximately 22 minutes to complete. As a reminder, your responses to this survey are critical to the success of this research in helping the sponsor design new products/services to help you support your needs. Your responses will be kept strictly confidential and only reported in combination with other respondents' data. In addition, you may be asked certain questions for quality control purposes.

ALL QUALIFIED RESPONDENTS (S100r1)
Q203 For the remainder of the survey, we would like to understand your experiences being diagnosed with, and treated for, Non-alcoholic steatohepatitis **— referred to as NASH.** We will ask about the time from when you first started experiencing symptoms, if at all, until today.

To the best of your knowledge, which of the following are the cause(s) of NASH?

Please select all that apply.

[RANDOMIZE, MULTIPLE SELECT]

1. Excess weight / being overweight
2. Genetics
3. Another condition I have been diagnosed with
4. Insulin resistance/Diabetes
5. Poor lifestyle habits (i.e., diet, exercise)
96. Other, please specify: [INSERT TEXT BOX. ANCHOR]
97. Not sure [EXCLUSIVE, ANCHOR]

ALL QUALIFIED RESPONDENTS (S100r1)
Q205 What NASH symptoms, if any, did you experience <u>prior</u> to your NASH diagnosis?

Please select all that apply.

[MULTPLE SELECT, RANDOMIZE]
1. Fatigue
2. Abdominal pain
3. Swelling in the abdomen
4. Loss of appetite
5. Bleeding/bruising easily
6. Itchy skin
7. Yellow discoloration in the skin and eyes (jaundice)
8. Nausea
9. Swelling in your legs
10. Slurred speech
11. Drowsiness
12. Chest pain
96. Other, please specify: [ANCHOR, MANDATORY TEXT BOX]
97. I didn't experience (or didn't realize I was experiencing) NASH symptoms until after I was diagnosed [EXCLUSIVE][ANCHOR]

[IF EXPERIENCED NASH SYMPTOMS (Q205rNE14) ASK Q206. ELSE SKIP TO Q227]

EXPERIENCED NASH SYMPTOMS PRIOR TO DIAGNOSIS (Q205rNE14)
Q206 At what age did you first experience NASH symptom(s)?
[RANGE: 0-S4]
1. |_|_| years old

EXPERIENCED NASH SYMPTOMS PRIOR TO DIAGNOSIS (Q205rNE14)
Q207 You mentioned that you first started experiencing NASH symptoms at [PIPE Q206] years old.

How long after you first started experiencing symptoms at age [PIPE Q206] did you **first discuss your symptoms with a healthcare provider**?

If less than one year, enter 0 for "years" and then only enter in the number of months. If less than one month, enter "0" for both "months" and "years."

1. |_|_| years [RANGE: 0-(S4-Q206)]
2. |_|_| months [RANGE: 0-11]

EXPERIENCED NASH SYMPTOMS PRIOR TO DIAGNOSIS (Q205rNE14)
Q207B BTS MONTHS CALCULATION

1. [INSERT MONTHS: Q207r1*12 + Q207r2]

EXPERIENCED NASH SYMPTOMS PRIOR TO DIAGNOSIS (Q205rNE14)
Q210 What caused you to discuss your NASH symptoms with a healthcare provider?

Please select all that apply.

[RANDOMIZE; MULTIPLE SELECT]
1. My symptoms worsened (in degree of discomfort/frequency/etc.)
2. My symptoms were interfering too much with my daily life
3. My symptoms were not resolving on their own
4. I was encouraged by family or friends
5. I started experiencing new symptoms that concerned me
6. My symptoms required immediate attention (e.g., severe pain)
7. My healthcare provider asked me about my symptoms
96. Other, please specify: [ANCHOR. INSERT TEXT BOX]

SYMPTOMS INTERFERED WITH DAILY LIFE (Q210r2)
Q210A You indicated that one of the reasons you discussed your NASH symptoms with a healthcare provider was <u>your symptoms were interfering too much with your daily life.</u>

Which of the following symptoms were interfering with your daily life?

Please select all that apply.

[MULTPLE SELECT, RANDOMIZE. SHOW ONLY THOSE SELECTED AT Q205]
1. Fatigue
2. Abdominal pain
3. Swelling in the abdomen
4. Loss of appetite
5. Bleeding/bruising easily
6. Itchy skin
7. Yellow discoloration in the skin and eyes (jaundice)
8. Nausea
9. Swelling in your legs
10. Slurred speech
11. Drowsiness
12. Chest pain
13. INSERT Q205r96

EXPERIENCED NASH SYMPTOMS PRIOR TO DIAGNOSIS (Q205rNE14)
Q215 For what reasons did you not bring up your symptoms with your healthcare provider sooner?

Please select all that apply.

[RANDOMIZE. MULTIPLE SELECT]
1. I didn't want to admit I was experiencing a health issue
2. I didn't think there was anything that my healthcare provider could do about it
3. I was afraid to find out what was going on
4. I thought my symptoms were due to another health condition
5. I was able to manage my symptoms with self-care methods (e.g., over-the-counter treatments, a change in physical activity or diet)
6. My symptoms did not limit my daily function and activities up until that point
96. Other, please specify: [ANCHOR. INSERT TEXT BOX]
97. I didn't wait to bring up my symptoms – it was the soonest I could get an appointment [EXCLUSIVE; ANCHOR]

EXPERIENCED NASH SYMPTOMS PRIOR TO DIAGNOSIS (Q205rNE14)
Q220 Which healthcare provider did you initially discuss your NASH symptoms with?

[ALPHA SORT; SINGLE SELECT]
1. Primary care physician (PCP/GP/FP/IM)
2. Hepatologist
3. Gastroenterologist
4. Endocrinologist
5. Obstetrician / Gynecologist (OB/GYN)
6. Cardiologist
7. Urgent Care/Emergency Room
8. Other, please specify: [ANCHOR; TEXT BOX]
9. I don't recall [ANCHOR]

EXPERIENCED NASH SYMPTOMS PRIOR TO DIAGNOSIS (Q205rNE14)
Q225 Prior to being formally diagnosed with NASH by a healthcare provider, how many healthcare providers did you see specifically because of your NASH symptoms?

If you were diagnosed with NASH at the first visit related to NASH symptoms, please enter "0."

[RANGE: 0-99]
1. |_|_| healthcare providers
2. Not sure

EXPERIENCED NASH SYMPTOMS PRIOR TO DIAGNOSIS (Q205rNE14)
Q226 What healthcare providers did you see specifically because of your NASH symptoms prior to your diagnosis?

Please select all that apply.
[ALPHA SORT; MULTI-SELECT; MUST SELECT Q220 RESPONSE IF r1-9]
1. Primary care physician (PCP/GP/FP/IM)
2. Hepatologist
3. Gastroenterologist
4. Endocrinologist
5. Obstetrician / Gynecologist (OB/GYN)
6. Cardiologist
7. Urgent Care/Emergency Room
8. INSERT Q220r8
9. Other, please specify: [ANCHOR; TEXT BOX]
10. I don't recall [ANCHOR]

ALL QUALIFIED RESPONDENTS (S100r1)
Q227 What specific tests were used to determine (diagnose) that you had NASH?

Please select all that apply.

[RANDOMIZE, MULTIPLE SELECT]
1. Liver function tests – *a group of blood tests intended to measure the presence of various liver enzymes*
2. Imaging – *potentially an MRI, MRE, VCTE, CT, or ultrasound, used to visualize organs inside the body (particularly your liver)*
3. FibroScan – *a specific form of medical imaging used to assess the 'hardness' or 'stiffness' of the liver*
4. Liver biopsy – *procedure in which a small needle is inserted into the liver to collect a tissue sample*
5. NASH biomarker panel
6. Other lab test, please specify [MANDATORY TEXT BOX, ANCHOR]
7. Not sure/Don't recall [EXCLUSIVE, ANCHOR]

ALL QUALIFIED RESPONDENTS
Q230 What healthcare provider(s) **ordered the tests** that eventually led to your NASH diagnosis? The healthcare provider(s) who ordered tests may or may not be the same who provided your diagnosis.

[ALPHA SORT; MULTI-SELECT]
1. Primary care physician (PCP/GP/FP/IM)
2. Hepatologist
3. Gastroenterologist
4. Endocrinologist
5. Obstetrician / Gynecologist (OB/GYN)
6. Cardiologist
7. Urgent Care/Emergency Room
8. INSERT Q220r8
9. INSERT Q226r9
10. Other, please specify: [ANCHOR; TEXT BOX]
11. I don't recall [ANCHOR]

ALL QUALIFIED RESPONDENTS
Q235 And, whether or not it was the same provider who ordered the diagnostic tests, what healthcare provider officially diagnosed you with NASH?

[ALPHA SORT; SINGLE-SELECT]
1. Primary care physician (PCP/GP/FP/IM)
2. Hepatologist
3. Gastroenterologist
4. Endocrinologist
5. Obstetrician / Gynecologist (OB/GYN)
6. Cardiologist
7. Urgent Care/Emergency Room
8. INSERT Q220r8
9. INSERT Q226r9
10. INSERT Q230r10
11. Other, please specify: [ANCHOR; TEXT BOX]
12. I don't recall [ANCHOR]

ALL QUALIFIED RESPONDENTS (S100r1)
QC1 For quality control purposes, please select no.
1. Yes
2. No

SECTION 300: PATHWAY

EXPERIENCED NASH SYMPTOMS PRIOR TO DIAGNOSIS (Q205rNE14)
Q300 You mentioned that you first started experiencing NASH symptoms at [PIPE Q206] years old.

How long after you first started experiencing symptoms at age [PIPE Q206r1] were you **formally diagnosed with NASH by a healthcare provider?**

If less than one year, enter 0 for "years" and then only enter in the number of months. If less than one month, enter "0" for both "months" and "years."

1. |_|_| years [RANGE: 0-(S4-Q206)]
2. |_|_| months [RANGE: 0-11]

ALL QUALIFIED RESPONDENTS (S100r1)
Q207D BTS MONTHS CALCULATION

2. [INSERT MONTHS: Q300Cr1*12 + Q300Cr2]

ALL QUALIFEID RESPONDENTS
Q305 When you were formally diagnosed with NASH, how did your healthcare provider characterize it to you?

1. My healthcare provider described it as Non-alcoholic steatohepatitis (NASH) [ANCHOR AT TOP]
2. My healthcare provider described it as Non-alcoholic steatohepatitis (NASH **with fibrosis**)
3. My healthcare provider described it as Non-alcoholic steatohepatitis (NASH **without fibrosis**)
2. My healthcare provider described it as Non-alcoholic fatty liver disease (NAFLD)
3. My healthcare provider described it as Fatty Liver disease
4. My healthcare provider described it in another way: [ANCHOR; TEXT BOX]
5. I don't know/don't remember

ALL QUALIFEID RESPONDENTS
Q310 You mentioned that you were diagnosed with NASH [S17] years ago.

How long after your diagnosis [PIPE S17] years ago were you **first treated specifically for NASH?**

By "treatment", we are referring to any medicines, therapies, regimens, or course of action prescribed or recommended.

If less than one year, enter 0 for "years" and then only enter in the number of months. If less than one month, enter "0" for both "months" and "years."

1. |_|_| years [RANGE: 0-(S17)]
2. |_|_| months [RANGE: 0-11]

ALL QUALIFIED RESPONDENTS (S100r1)
Q310F BTS MONTHS CALCULATION

3. [INSERT MONTHS: Q310r1*12 + Q310Er2]

ALL QUALIFEID RESPONDENTS
Q315 Now, we want to know more about the types of healthcare providers you've seen for NASH over time **-** from the first time an issue was suspected or discussion of symptoms, until today.

What specialties of healthcare providers (or the specialty of their office/clinic) have you **ever** seen for NASH?

Please consider all providers you saw (regardless of the initial reason) so long as seeing them was related to NASH (i.e., initial evaluation, diagnosis, ongoing treatment and management). We're interested in those you have ever seen, even if you are not seeing them any longer.

[RANDOMIZE, GROUP CODES 2/3; MULTIPLE SELECT; FORCE Q226 RESPONSE]

1. Primary care physician (PCP/GP/FP/IM)
2. Hepatologist
3. Gastroenterologist
4. Endocrinologist
5. Obstetrician / Gynecologist (OB/GYN)
6. Cardiologist
7. Urgent Care/Emergency Room
8. INSERT Q220r8
9. INSERT Q226r96
96. Other, please specify: [MANDATORY TEXT BOX. ANCHOR]

ALL QUALIFIED RESPONDENTS (S100r1)
Q320 Do you remember what type of healthcare provider first told you your symptoms and/or test results might be NASH (i.e., used the term "NASH" or said they suspected you had NASH), regardless of whether they confirmed your diagnosis?

1. Yes
2. No

REMEMBERS PROVIDER (Q320r1)
Q325 What specialty of healthcare provider first told you your symptoms and/or test results might be NASH (i.e., used the term "NASH" or said they suspected you had NASH), regardless of whether they confirmed your diagnosis?

[RANDOMIZE; ONLY SHOW THOSE SELECTED AT Q315]

1. Primary care physician (PCP/GP/FP/IM)
2. Hepatologist
3. Gastroenterologist
4. Endocrinologist
5. Obstetrician / Gynecologist (OB/GYN)
6. Cardiologist
7. Urgent Care/Emergency Room
8. INSERT Q220r8

9. INSERT Q226r96
96. Other, please specify: [MANDATORY TEXT BOX. ANCHOR]
97. I don't recall [EXCLUSIVE, ANCHOR]

ALL QUALIFIED RESPONDENTS (S100r1)
Q335 How many of each of the following specialties of provider have you **ever** seen for NASH?

Again, please consider all providers you saw (regardless of the reason) so long as seeing them was related to NASH *(i.e., initial evaluation, diagnosis, ongoing treatment and management). If you saw both a physician at the practice and a Nurse practitioner/Physician Assistant (NP/PA), please consider them separate individuals)*

[COLUMN]
1. Number of providers

[ROWS, ONLY SHOW THOSE SELECTED AT Q315 IN SAME ORDER; RANGE 1-99]

1. Primary care physician (PCP/GP/FP/IM)	|_|_|_|
2. Hepatologist	|_|_|_|
3. Gastroenterologist	|_|_|_|
4. Endocrinologist	|_|_|_|
5. Obstetrician / Gynecologist (OB/GYN)	|_|_|_|
6. Cardiologist	|_|_|_|
7. Urgent Care/Emergency Room	|_|_|_|
8. [INSERT Q315r96 RESPONSE]	|_|_|_|

ALL QUALIFIED RESPONDENTS (S100r1)
Q335A HIDDEN QUESTION – NUMBER OF PROVIDER TYPES SEEN

[SELECT PROVIDERS BELOW BASED ON NUMERIC ENTRY AT Q335]
1. Primary care physician (PCP/GP/FP/IM) 1
2. Primary care physician (PCP/GP/FP/IM) 2
3. Hepatologist 1
4. Hepatologist 2
5. Gastroenterologist 1
6. Gastroenterologist 2
7. Endocrinologist 1
8. Endocrinologist 2
9. Obstetrician / Gynecologist (OB/GYN) 1
10. Obstetrician / Gynecologist (OB/GYN) 2
11. Cardiologist 1
12. Cardiologist 2
13. Urgent Care/Emergency Room 1
14. Urgent Care/Emergency Room 2
15. [INSERT Q315r96 RESPONSE] 1
16. [INSERT Q315r96 RESPONSE] 2

ALL QUALIFIED RESPONDENTS (S100r1)

Q335B Now, for each of the following providers, please enter their initials – we will refer to providers in the questions that follow using their initials. For example, Dr. Anne Smith would be “A.S.” If more than one provider has the same initials, please make a unique identifier to differentiate – for example, “A. Smith.”

Please note: from this point on we will only ask you about up to 2 providers of a particular specialty, even if you have seen more than 2. If you've seen more than 2:

- ***Provider 1 should be the first provider*** *you saw of a particular specialty*
- ***Provider 2 should be*** *another provider* ***– whichever has been most influential on your NASH care***.

For example, if you saw three Gastroenterologists, you would enter the initials of the 1st Gastroenterologist you saw for NASH as Gastroenterologist 1, and for Gastroenterologist 2 you would enter the initials of one of the other two Gastroenterologists you've seen – whichever has been most influential on your NASH care.

[COLUMNS]

1. Initials (or other unique identifier) |_|_|_|

[ROWS; INSERT BASED ON Q335A]

1. Primary care physician (PCP/GP/FP/IM) 1
2. Primary care physician (PCP/GP/FP/IM) 2
3. Hepatologist 1
4. Hepatologist 2
5. Gastroenterologist 1
6. Gastroenterologist 2
7. Endocrinologist 1
8. Endocrinologist 2
9. Obstetrician / Gynecologist (OB/GYN) 1
10. Obstetrician / Gynecologist (OB/GYN) 2
11. Cardiologist 1
12. Cardiologist 2
13. Urgent Care/Emergency Room 1
14. Urgent Care/Emergency Room 2
15. [INSERT Q315r96 RESPONSE] 1
16. [INSERT Q315r96 RESPONSE] 2

[FOR QUESTIONS BELOW REQUIRING PIPING OF PROVIDER, DISPLAY AS ROW LABEL AND INITIALS FROM Q335Bc1 IN PARENTHESES, BUT CODE AS ROW LABEL]

ALL QUALIFIED RESPONDENTS (S100r1)

Q340 Now, please place the providers you have seen in chronological order starting with the type of healthcare provider you saw first, the type you saw next, etc.

If you indicated you saw more than one of a provider type, multiple options for each provider type will be available – for example, if you saw two Gastroenterologists, “Gastroenterologist 1” will be the first Gastroenterologist you saw. If you saw more than two of a provider type, please use the 2nd provider category to indicate the most recent provider of that type seen.

[SHOW RELEVANT SELECTION FROM Q335A USING ROW LABEL AND INITIALS FROM Q335B IN PARENTHESES. DRAG AND DROP RANK ORDER]

ALL QUALIFIED RESPONDENTS (S100r1)
Q345 For what reason(s) have you ever seen the following providers related to your NASH care?

Please select all that apply.

You may only select one provider for "diagnosis" and "initial treatment."

[FORCE RESPONSE FOR EACH ROW. FORCE RESPONSE FOR C2 AND C4, DO NOT FORCE RESPONSE FOR ALL OTHER COLUMNS]

[COLUMNS]

1. Initial symptom evaluation <u>(prior to diagnosis)</u>
2. Diagnosis of NASH [ONLY ALLOW 1 SELECTION]
3. Second opinion for diagnosis
4. Initial treatment for NASH [ONLY ALLOW 1 SELECTION]
5. Ongoing treatment and management of NASH
6. Comorbidity management <u>related to NASH</u>

[ROWS]
[SHOW SELECTIONS FROM Q335A IN Q340 ORDER WITH ROW LABEL AND Q335B INITIALS IN PARENTHESES]

[LOOP Q350-Q365 FOR ALL PROVIDERS SEEN AT Q335A IN Q340 ORDER WITH ROW LABEL AND Q335B INITIALS IN PARENTHESES]

ALL QUALIFIED RESPONDENTS (S100r1)
Q350 Is this provider a physician?

By physician we mean a medical doctor (MD) rather than a nurse practitioner (NP) or physician assistant (PA)

1. Yes
2. No
3. I am not sure / do not recall

ALL QUALIFIED RESPONDENTS (S100r1)
Q355 Are you still seeing [INSERT ROW LABEL (INSERT Q335B PROVIDER INITIALS)]?

1. Yes
2. No

DOESN'T STILL SEE HCP (Q355r2)
Q360 For what reasons do you no longer see [INSERT ROW LABEL (INSERT Q335B PROVIDER INITIALS)]?

Please select all that apply.

[RANDOMIZE. MULTIPLE SELECT]

1. I moved away from the area they are in
2. Did not feel they were helpful/necessary
3. No follow-up visit was scheduled / recommended by provider
4. They did all they could to help, but it wasn't enough
5. Started seeing another type of provider
6. My insurance coverage changed
7. They moved/retired
8. I did not like them/we were not a good match

96. Other, please specify: [INSERT TEXT BOX, ANCHOR]

[SHOW Q361-Q363B ON SAME SCREEN]
ALL QUALIFIED RESPONDENTS (S100r1)
Q361 Is [INSERT ROW LABEL (INSERT Q335B PROVIDER INITIALS)…?

Please select all that apply.

[MULTIPLE SELECT]

1. White
2. Black or African American
3. Spanish/Hispanic or Latino
4. American Indian or Alaska Native
5. Asian (i.e., Far East, Southeast Asia, or the Indian subcontinent)
6. Other, please specify: ________
7. Decline to answer [EXCLUSIVE]

ALL QUALIFIED RESPONDENTS (S100r1)
Q362 How would you describe [INSERT ROW LABEL (INSERT Q335B PROVIDER INITIALS)?

1. Male
2. Female
3. Transgender
4. Do not identify as female, male or transgender
5. Decline to answer

ALL QUALIFIED RESPONDENTS (S100r1)
Q363 How old is [INSERT ROW LABEL (INSERT Q335B PROVIDER INITIALS) in relation to your age?

1. Younger than me
2. Around the same age as me
3. Older than me
4. Decline to answer

ALL QUALIFIED RESPONDENTS (S100r1)
Q363A Which best describes the location of [INSERT ROW LABEL (INSERT Q335B PROVIDER INITIALS)'s practice?

1. Urban
2. Suburban
3. Rural
4. Decline to answer

ALL QUALIFIED RESPONDENTS (S100r1)
Q363B Which of the following best describes [INSERT ROW LABEL (INSERT Q335B PROVIDER INITIALS)'s type of practice?

1. Hospital
2. Private solo practice (only one doctor)
3. Hospital affiliated practice
4. Private group practice (multiple doctors)
5. Ambulatory surgery center
6. Government or VA hospital
7. Urgent care facility
8. Walk-in clinic (Minute Clinic, Healthcare Clinic, RediClinic, etc.)
9. Other, please specify: [ANCHOR; TEXT BOX]
10. Not sure

[LOOP FOR ALL Q335A PROVIDERS IN Q340 ORDER]

[END OF LOOP]

ALL QUALIFIED RESPONDENTS (S100r1)

Q365 Now, we'd like to learn a little more about the treatments you have used for NASH.

Which of the following treatments have you **ever** been prescribed or recommended by a healthcare provider and used for treatment of your NASH symptoms, and which are you **currently** using?

[COLUMNS]

5. I am currently using this treatment/approach
6. I used this treatment/approach in the past, but I am not currently using it
7. I have never used this treatment/approach

[ROWS. MULTIPLE SELECT. RANDOMIZE]

1. Lifestyle changes which focus on exercise (e.g., increasing physical activity)
2. Lifestyle changes which focus on diet (e.g., using a specific diet or diet program (Jenny Craig, Weight Watchers, Elimination diets, Keto, Medifast, reducing calories, salt, sugar)
3. Prescription type 2 diabetes medications (pioglitazone/Actos, metformin, GLP-1s like Victoza, Ozempic)
4. Prescription weight loss medication (e.g., Saxenda, Xenical, Qsymia, Contrave)
5. Limiting or abstaining from alcohol
6. Bariatric surgery
7. Obeticholic acid (prescribed by healthcare provider)
8. Supplements like vitamin E or milk thistle

96. Other, please specify: [INSERT TEXT BOX. ANCHOR]
97. I have never been treated for my NASH symptoms [ANCHOR; EXCLUSIVE]

[IF HAS EVER BEEN TREATED FOR NASH (Q365r1-96) ASK Q370. ELSE JUMP TO Q400.]

HAS EVER BEEN TREATED FOR NASH (Q365r1-96)

Q370 Which of the following treatments prescribed or recommended by a healthcare provider did you use prior to your diagnosis of NASH?

Please only consider treatment initiated or suggested by a healthcare provider.

Please select all that apply.

[ROWS. MULTIPLE SELECT. RANDOMIZE. ONLY SHOW TREATMENTS SELECTED AT Q365C1 AND C2]

1. Lifestyle changes which focus on exercise (e.g., increasing physical activity)
2. Lifestyle changes which focus on diet (e.g., using a specific diet or diet program (Jenny Craig, Weight Watchers, Elimination diets, Keto, Medifast, reducing calories, salt, sugar)
3. Prescription type 2 diabetes medications (pioglitazone/Actos, metformin, GLP-1s like Victoza, Ozempic)
4. Prescription weight loss medication (e.g., Saxenda, Xenical, Qsymia, Contrave)
5. Limiting or abstaining from alcohol
6. Bariatric surgery
7. Obeticholic acid (prescribed by healthcare provider)
8. Supplements like vitamin E or milk thistle

96. Other, please specify: [INSERT TEXT BOX. ANCHOR]
97. I have never been treated prior to my NASH diagnosis [ANCHOR; EXCLUSIVE]

ALL QUALIFIED RESPONDENTS (S100r1)

QC3 For quality control purposes, please select “5” from the list of options below.

1. 1
2. 2
3. 3
4. 4
5. 5

SECTION 400: DIAGNOSIS

ALL QUALIFIED RESPONDENTS (S100r1)
Q400 Now that we understand more about your experience with NASH overall, we want to know more about some specific points in time related to your diagnosis and ongoing care. First, we want to learn a little more about your experiences **being diagnosed with NASH**.

Which of the following <u>best describes</u> how you were formally diagnosed with NASH?

1. I was diagnosed at a well-visit or annual exam
2. I was diagnosed at an appointment made specifically to discuss my NASH symptoms
3. I was diagnosed at an appointment made specifically to discuss formal testing for suspected NASH
4. I was diagnosed at an appointment for another condition where NASH was discovered incidentally when reviewing test results
5. I was diagnosed at the Emergency Room after a sudden onset of symptoms
96. Other, please specify: [MANDATORY TEXT BOX][ANCHOR]
97. I do not recall [ANCHOR]

ALL QUALIFIED RESPONDENTS (S100r1)
Q401 <u>When you were first diagnosed with NASH</u>, which of the following did your **diagnosing provider** talk to you about?

Please select all that apply.

[RANDOMIZE; MULTIPLE SELECT]
1. Causes of NASH
2. Treatments for NASH (e.g., lifestyle modifications, management of other medical conditions)
3. How NASH is related to, or impacts, my other health conditions
4. Progression of NASH to fibrosis
5. Progression of NASH to cirrhosis
6. Progression of NASH to liver cancer
7. Progression of NASH to the point of needing a liver transplant
8. Regression of NASH (ability to reverse the condition)
96. Other, please specify: [INSERT TEXT BOX. ANCHOR]
97. I do not recall [EXCLUSIVE. ANCHOR]
98. None of these [EXCLUSIVE. ANCHOR]

ALL QUALIFIED RESPONDENTS (S100r1)

Q402 <u>When you were first diagnosed with NASH</u>, what, if any, resources did your **diagnosing provider** provide to help with your understanding of NASH or how to manage/treat it?

Please select all that apply.

[MULTIPLE SELECT]

1. Reading material (i.e., pamphlet/brochure, book, magazine) about the condition
2. Website
3. Information on local support group
4. Information on advocacy groups
5. Smartphone app

96. Other, please specify: [INSERT TEXT BOX. ANCHOR]
97. My healthcare provider did not provide me with any resources at this time [EXCLUSIVE. ANCHOR]
98. I do not recall [EXCLUSIVE. ANCHOR]

[IF PROVIDED WEBSITE (Q320r2) ASK Q321. ELSE SKIP TO PN AFTER Q321.]

PROVIDED WEBSITE (Q402r2)
Q403 You mentioned that your **diagnosing provider** provided you with website(s) to help with your understanding of NASH or how to manage/treat it. What specific websites did they provide you with?

Please enter all websites you were provided.

[SHOW 1 ANSER TEXT BOX] ONCE TEXT IS ENTERED SHOW NEXT]

11. MANDATORY TEXT BOX
12. MANDATORY TEXT BOX
13. MANDATORY TEXT BOX

[IF PROVIDED SUPPORT GROUP (Q402r3-4) ASK Q404. ELSE SKIP TO PN AFTER Q404]

PROVIDED SUPPORT GROUP OR ADVOCACY (Q402r3-4)
Q404 You mentioned that your **diagnosing provider** provided you with information on local support group(s) and/or advocacy groups to help with your understanding of NASH or how to manage/treat it. What specific support group(s) did they provide you with?

Please enter all groups you were provided.

[SHOW 1 ANSER TEXT BOX] ONCE TEXT IS ENTERED SHOW NEXT]

1. MANDATORY TEXT BOX
2. MANDATORY TEXT BOX
3. MANDATORY TEXT BOX

ALL QUALIFIED RESPONDENTS (S100r1)
Q405 <u>When you were first diagnosed with NASH,</u> did your **diagnosing provider** specifically discuss **lifestyle modification** with you?

1. Yes
2. No
3. I don't recall

[IF DISCUSSED LIFESTYLE MODIFICATION (Q405r1) ASK Q406. ELSE SKIP TO Q400A]

DISCUSSED LIFESTYLE MODIFICATION (Q405r1)
Q406 When you were first diagnosed with NASH, which, if any, of the following did your **diagnosing provider** discuss with you?

Please select all that apply. Please consider only the things your healthcare provider did/discussed when you were initially diagnosed, not anything that they may have recommended later in your care.

[MULTIPLE SELECT. RANDOMIZE]
1. Explain the effect my weight has on my NASH
2. Explain the effect my NASH has on my weight
3. Explain the effect exercise has on my NASH
4. Help me set goals to improve my NASH
5. Help me understand the effect of high lipid levels on my NASH
6. Help me understand the effect of carbohydrates on my NASH
7. Help me understand the effect of sugars on my NASH
8. Make me aware of medications that will help me lose weight
9. Suggest I see a registered Dietitian or Nutritionist
96. Other, please specify: [INSERT TEXT BOX. ANCHOR]
97. I do not recall [ANCHOR. EXCLUSIVE]

SECTION 500: ONGOING MANAGEMENT

ALL QUALIFIED RESPONDENTS (S100r1)
Q500A Now, we'd like to learn a little more about the ongoing treatment and management of your NASH.

Please click to continue.

ALL QUALIFIED RESPONDENTS (S100r1)
Q505 Below are the providers you have seen for NASH treatment and management. Please select the two providers that have been **most influential** in the treatment and management of your NASH.

Please select two.

[RANDOMIZE; IF ONLY ONE RESPONSE AVAILABLE AUTOFILL AND SKIP]

1. [SHOW SELECTIONS FROM Q335A WITH ROW LABEL AND Q335B INITIALS IN PARENTHESES]

[LOOP Q415-Q440 THROUGH TWO PROVIDERS SELECTED AT Q505. PIPED TEXT NEEDS TO BE ROW LABEL AND Q335B INITIALS IN PARENTHESES. IF NO PROVIDERS AVAILABLE TO SELECT AT Q505, SKIP TO Q545]

ALL QUALIFIED RESPONDENTS (S100r1)

Q508 For the next several questions, we'd like you to think about [INSERT Q505], and how this healthcare provider has helped you manage your NASH over time.

First, please indicate how much you agree with each of the following statements regarding **why** you consider [INSERT Q405] to be influential in the treatment and management of your NASH.

I find [INSERT Q405] influential in the treatment and management of my NASH because….

Please use the scale below where '1' indicates "Do not agree at all," and '7' indicates "Completely agree"

[COLUMNS]

Do not agree at all						Completely agree
1	2	3	4	5	6	7

[ROWS; RANDOMIZE; SHOW AS CAROUSEL]

1. He/she provides me with all possible treatment options
2. He/she provides ongoing support
3. He/she has a similar background as me (i.e., socioeconomic)
4. He/she has a similar cultural background as me (i.e., ethnicity)
5. The location of the practice he/she works at
6. I trust their advice
7. Their age in relation to my own
8. I feel he/she can relate to me

ALL QUALIFIED RESPONDENTS (S100r1)
Q510 How frequently do you (or did you) typically have appointments with [INSERT Q505] **where your NASH is discussed**?

1. Weekly
2. Monthly
3. Every other month
4. Every 3 months
5. Every 6 months
6. Yearly
96. Other, please specify: [INSERT TEXT BOX]

ALL QUALIFIED RESPONDENTS (S100r1)
Q515 Which of the following topics has [INSERT Q505] **ever** discussed with you?

Please select all that apply.

[RANDOMIZE; MULTIPLE SELECT]

1. Causes of NASH
2. Treatments for NASH (e.g., lifestyle modifications, management of other medical conditions)
3. How NASH is related to, or impacts, my other health conditions
4. Progression of NASH (what to expect in the future)
5. Regression of NASH (ability to reverse the condition)
96. Other, please specify: [INSERT TEXT BOX. ANCHO]

97. I do not recall [EXCLUSIVE. ANCHOR]

ALL QUALIFIED RESPONDENTS (S100r1)
Q520 Which resources has [INSERT Q505] **ever** provided to you to help with your understanding or management of NASH?

Please select all that apply.

[MULTIPLE SELECT]
1. Reading material (i.e., pamphlet/brochure, book, magazine) about the condition
2. Website
3. Information on local support group
4. Information on advocacy groups
5. Smartphone app
96. Other, please specify: [INSERT TEXT BOX][ANCHOR]
97. My healthcare provider has not provided me with any resources [EXCLUSIVE]
98. I do not recall [EXCLUSIVE. ANCHOR]

[IF PROVIDED WEBSITE (Q520r2) ASK Q525. ELSE SKIP TO PN AFTER Q525.]

PROVIDED WEBSITE (Q520r2)
Q525 You mentioned that [INSERT Q505] provided you with website(s) to help with your understanding of NASH or how to manage/treat it. What specific websites did they provide you with?

Please enter all websites you were provided.

[SHOW 1 ANSER TEXT BOX] ONCE TEXT IS ENTERED SHOW NEXT]
1. MANDATORY TEXT BOX
2. MANDATORY TEXT BOX
3. MANDATORY TEXT BOX

[IF PROVIDED SUPPORT GROUP (Q520r3) ASK Q432. ELSE SKIP TO Q436.]

PROVIDED SUPPORT GROUP OR ADVOCACY GROUP (Q520r3-4)
Q530 You mentioned that [INSERT Q505] provided you with information on local support and/or advocacy group(s) to help with your understanding of NASH or how to manage/treat it. What specific support group(s) did they provide you with?

Please enter all groups you were provided.

[SHOW 1 ANSER TEXT BOX] ONCE TEXT IS ENTERED SHOW NEXT]
1. MANDATORY TEXT BOX
2. MANDATORY TEXT BOX
3. MANDATORY TEXT BOX

ALL QUALIFIED RESPONDENTS (S100r1)

Q535 About how frequently (if at all) does [INSERT Q505] discuss lifestyle modification with you?

1. At every appointment
2. At most appointments
3. At some appointments
4. Rarely / at very few appointments
5. Never

HAS DISCUSSED LIFESTYLE MODIFICATION (Q535r1-4)
Q540 When [INSERT Q505] discusses lifestyle modification with you **during ongoing treatment and management**, which of the following have they discussed?

Please select all that apply.

[MULTIPLE SELECT. RANDOMIZE]
1. Explain the effect my weight has on my NASH
2. Explain the effect my NASH has on my weight
3. Help me set goals to improve my weight
4. Help me understand why I have excess weight
5. Make me aware of medications that will help me lose weight
6. Tell me about ways to lose weight (i.e., programs (e.g., Jenny Craig), diet, exercise)
7. Tell me about weight loss clinics
8. Suggest I see a registered Dietitian or Nutritionist
98. Other, please specify: [INSERT TEXT BOX. ANCHOR]
99. I do not recall [ANCHOR. EXCLUSIVE]

[LOOP THROUGH ALL Q505 REMAINING HCPS]

[END LOOP]

ALL QUALIFIED RESPONDENTS (S100r1)
Q545 Which type of healthcare provider do you consider the "**coordinator" of your NASH care**?

By "coordinator" please consider the healthcare provider who is primarily responsible for managing your NASH in conjunction with any other conditions you have.

1. [INSERT Q335B PROVIDER LIST WITH INITIALS]
96. Other, please specify: [INSERT TEXT BOX]
97. I don't consider any healthcare providers to be the coordinator of my care [EXCLUSIVE]

ALL QUALIFIED RESPONDENTS (S100r1)
Q550 What are the biggest motivators for you in managing your NASH?

Please select up to 3 motivators.

[MULTISELECT SELECT 3; RANDOMIZE]
1. Improving my NASH

2. Living longer
3. Enjoying life to the fullest
4. Spending more time with my family
5. Improving my other medical conditions
6. Making my healthcare provider proud
7. Being able to participate in activities I am not currently able to
8. Having fewer illnesses/conditions to worry about
96. Other, please specify: [INSERT TEXT BOX, ANCHOR]
97. I don’t have any drivers in managing my NASH [EXCLUSIVE. ANCHOR]

ALL QUALIFIED RESPONDENTS (S100r1)
Q560 What would indicate that your NASH treatment and management has been a success?

Please select up to 3 successes.
[MULTISELECT SELECT 3; RANDOMIZE]
1. Improvement in LFTs/bloodwork
2. Improvement in symptoms (if applicable)
3. Improvement in my other conditions
4. Less frequent visits to the doctor for check-ups
5. NASH biomarker panel normalization
6. Reverting (decrease in severity) my NASH
7. Resolving (getting rid of) my NASH
8. Prevention of cirrhosis
9. Not needing a liver transplant
10. Other, please specify: [ANCHOR; TEXT BOX]
11.

SECTION 600: IMPACT OF NASH AND COMORBIDITIES

ALL QUALIFIED RESPONDENTS (S100r1)

Q600 Now we would like to understand how NASH impacts your life. How much would you say your NASH (and any associated symptoms or requirements for managing it) impacts each of the following aspects of your daily life?

Please use a scale from 1 to 7 where "1" means it "Doesn't impact at all" and "7" means "Greatly impacts."

[RANDOMIZE ROWS]	1 – Doesn't impact at all	2	3	4	5	6	7 – Greatly impacts
1. Your social relationships and activities							
2. Your family relationships and activities							
3. Your finances							
4. Doing household chores (cooking, cleaning, etc.)							
5. Running errands (go to the grocery store, doctors' appointments, etc.)							
6. Your job or career							
7. Being active / exercising							
8. Your hobbies							
9. Your mental / emotional health							

ALL QUALIFIED RESPONDENTS (S100r1)
Q605 How much would you say the **other conditions you are diagnosed with** impact the cause, progression, and severity of your NASH? Please answer based on what you've heard or what you personally think.

Please use a scale from 1 to 7 where "1" means it "Doesn't impact at all" and "7" means "Greatly impacts."

[RANDOMIZE ROWS]	1 – Doesn't impact at all	2	3	4	5	6	7 – Greatly impacts
1. The cause of my NASH							
2. The progression of my NASH							
3. The severity of my NASH							

ALL QUALIFIED RESPONDENTS (S100r1)
Q607 When you were answering the previous questions concerning the impact your other conditions have on the cause, progression, and severity of your NASH, what other specific conditions were you thinking about?

Please select all that apply.
[ALPHA SORT. MULTIPLE SELECT; ONLY SHOW THOSE SELECTED IN S14; IF ONLY S14r1 SELECTED AUTO SELECT R21]]

1. [DO NOT SHOW]

2. Non-alcoholic fatty liver disease (NAFLD) / Fatty Liver Disease
3. Hepatitis B or C
4. Autoimmune hepatitis
5. Sleep apnea
6. Anxiety
7. Obesity
8. Type 1 diabetes
9. Type 2 diabetes
10. Pre-diabetes (which is a slightly elevated blood glucose levels, regarded as indicative that a person is at risk of progressing to Type 2 diabetes)
11. Cardiovascular / heart disease (including heart failure)
12. Depression
13. Hypertension (high blood pressure)
14. Osteoarthritis (OA)
15. Dyslipidemia (high cholesterol)
16. Chronic obstructive pulmonary disease (COPD)
17. Hypertriglyceridemia (high triglycerides)
18. Cancer
19. PCOS (Polycystic ovary syndrome) and infertility in women
20. INSERT S14r96
21. None of the above [ANCHOR. EXCLUSIVE]

ALL QUALIFIED RESPONDENTS (S100r1)
Q608 Please indicate how much you agree with each of the following statements regarding your NASH and the other conditions you are diagnosed with.

Please use a scale from 1 to 7 where "1" means it "Not at all agree" and "7" means "Completely agree."

[RANDOMIZE ROWS]	1 – Not at all agree	2	3	4	5	6	7 – Completely agree
1. I am motivated to treat my other conditions because doing so will improve my NASH							
2. I am always compliant with my treatments for the conditions I am diagnosed with							
3. It is my healthcare provider's responsibility to treat my NASH							
4. It is my responsibility to treat my NASH							
5. There is nothing I can do to improve my NASH							
6. Medication is the only way I am willing to treat my NASH							
7. My doctors don't seem to pay as much attention to my NASH as my other conditions							

8. It is my own fault that I have NASH							

ALL QUALIFIED RESPONDENTS (S100r1)
Q610 Which of the following methods have you **ever** used as a way to manage the other conditions you are diagnosed with (not NASH), and which are you **currently** using?

Please select all that apply.

[COLUMNS; MULTISELECT]
1. Ever used
2. Currently using

[ROWS; RANDOMIZE]
1. Exercise
2. Diet
3. Prescription medications
4. Over-The-Counter (OTC) medications
5. Surgery
96. Other
99. I have never used any methods to manage my other comorbidities [ANCHOR; EXCLUSIVE]
100. I am not currently using any methods to manage my other comorbidities [ANCHOR; EXCLUSIVE FOR COLUMN 2]

[IF EVER USED LIFESTYLE MODIFICATION BUT NOT CURRENTLY (Q610R1-2C1 AND Q610R1-2CNE2] ASK Q511. ELSE SKIP TO PN AFTER Q511]

IF EVER USED LIFESTYLE MODIFICATION BUT NOT CURRENTLY (Q610R1-2C1 AND Q610R1-2CNE2)
Q611 You indicated that you have used lifestyle modification (diet and/or exercise) in the past as a way to manage the other conditions you are diagnosed with (not NASH). For what reasons did you stop diet and/or exercise?

Please be as detailed as possible.

[LARGE MANDATORY TEXT BOX]

[IF EVER USED MEDICATION BUT NOT CURRENTLY (Q610R5C1 AND Q610R5CNE2] ASK Q612. ELSE SKIP TO AFTER Q612]

EVER USED MEDICATION BUT NOT CURRENTLY (Q610R3-4C1 AND Q610R3-4CNE2)
Q612 You indicated that you have used medication in the past as a way to manage the other conditions you are diagnosed with (not NASH). For what reasons did you stop using medication?

Please be as detailed as possible.

[LARGE MANDATORY TEXT BOX]

SECTION 700: SUPPORT AND RESOURCES

HAS SEEN MULTIPLE PROVIDERS WHO DISCUSSED COMORBIDITIES (>1 PROVIDER SELECTED FROM Q345r6]
Q700 Now that we've learned about the healthcare providers you have seen, thinking across those who have discussed the management of your other medical conditions (besides NASH) with you, which do you feel has been the <u>most</u> helpful in supporting you in managing lifestyle modification?

Please select only one.

1. [SHOW ONLY THOSE SELECTED AT Q345r6]
2. Other, please specify: [ANCHOR; TEXT BOX]
97. None of these providers have been helpful

PROVIDER HAS BEEN HELPFUL (Q700NE97)
Q705 For what reasons do you feel [INSERT Q600] is the most helpful in supporting you in managing lifestyle modification? What do they do that is helpful?

Please be as specific as possible.

[LARGE MANDATORY TEXT BOX]

ALL QUALIFIED RESPONDENTS (S100r1)
Q710 Which of the following have you used as a source of information related to <u>management of NASH and/or your other medical conditions</u>?

Please select all that apply.

[RANDOM, MULTI-SELECT]

1. The Internet (Google, Social media, Websites)
2. Family and friends
3. Information from a healthcare provider
4. Wellness coach or personal trainer
5. Registered Dietitian or Nutritionist (non-physician)
6. Peer support group
7. Patient advocacy groups
8. Smartphone apps
9. None of the above [ANCHOR, EXCLUSIVE]

USES INTERNET (Q710r1)

Q715 What specific websites do you consult for information on management of NASH and/or your other medical conditions?

[MANDATORY TEXT BOX]

USES PEER OR ADVOCACY GROUPS (Q710r6-7)
Q717 What specific support and/or advocacy groups do you consult for information on management of NASH and/or your other medical conditions?

[MANDATORY TEXT BOX]

ALL QUALIFIED RESPONDENTS (S100r1)
Q720 How would you most prefer to learn about NASH, whether you have used this source in the past or not?

*Please select your **top three** sources.*

[MULTIPLE SELECT. MAX 3 TO SELECT]
1. The Internet (Google, Social media, Websites)
2. Family and friends
3. Information from a healthcare provider
4. Wellness coach or personal trainer
7. Registered Dietitian or nutritionist (non-physician)
8. Peer support group
9. Patient advocacy groups
10. Smartphone apps
11. Other

RANKED OTHER SOURCE (Q720r11 RANKED)
QQ720A What is the other source you would prefer to use to learn about NASH and/or comorbidities?

[MANDATORY TEXT BOX]

ALL QUALIFIED RESPONDENTS (S100r1)
Q725 What are the **top 5 types of support that would be most helpful for you personally** to be successful with managing your NASH and other medical conditions?

Please select your top 5.

[SELECT 5 ONLY] [RANDOMIZE]
1. Resources for family and friends to help understand how to be supportive
2. Specific meal plans to follow for lifestyle management
3. Support groups (online or in-person)
4. Patient advocacy groups (online or in-person)
5. Programs offered at workplaces

6. Financial support for healthy choices
7. Wellness apps
8. Weekly follow-up visits with a healthcare provider
9. Access to a physician who specializes in NASH
96. Other, please specify: [INSERT TEXT BOX. ANCHOR]
97. I don't need any type of support [EXCLUSIVE, ANCHOR]

THOSE WHO WOULD FIND SUPPORT HELPFUL (Q725rNE97)
Q730 Please think about your personal NASH journey from before diagnosis to present day. Thinking about the top 5 types of support which would be most helpful to you, when along your journey would have they been most helpful for you?

[COLUMNS.ALLOW MULTI-SELECT]
1. Before diagnosis
2. When NASH was first suspected
3. When I was diagnosed with NASH
4. During treatment

[ROWS. SHOW THOSE ANSWERED IN Q725]
1. Resources for family and friends to help understand how to be supportive
2. Specific meal plans to follow for lifestyle management
3. Support groups (online or in-person)
4. Patient advocacy groups (online or in-person)
5. Programs offered at workplaces
6. Financial support for healthy choices (gym membership, healthy foods)
7. Wellness apps
8. Weekly follow-up visits with a healthcare provider or Dietitian/Nutritionist
9. Access to a physician who specializes in obesity
10. [INSERT Q725r96]

SECTION 800: CLINICAL TRIALS

ALL QUALIFIED RESPONDENTS (S100r1)
Q800 How interested would you be in using a pharmaceutical treatment (something prescribed by your doctor) for NASH?

Use a scale where a "1" indicates "Not at all interested," and a "7" indicates "Extremely interested."

[COLUMNS]

Not at all interested 1	2	3	4	5	6	Extremely interested 7

ALL QUALIFIED RESPONDENTS (S100r1)
Q805 What, if anything, would you need to know to feel comfortable using a pharmaceutical treatment to assist in improving your NASH?

Please be as detailed as possible.

[MANDATORY TEXT BOX[

ALL QUALIFIED RESPONDENTS (S100r1)
Q810 How interested would you be in participating in a clinical trial investigating the use of a pharmaceutical treatment to assist in the improvement of NASH?

Use a scale where a "1" indicates "Not at all interested," and a "7" indicates "Extremely interested."

Not at all interested 1	2	3	4	5	6	Extremely interested 7

ALL QUALIFIED RESPONDENTS (S100r1)
Q815 Now, we would like you to imagine that a new pharmaceutical treatment was available for NASH. What would your ideal mode of administration and frequency with which you had to take the treatment.

[SINGLE SELECT PER COLUMN]

**Mode of Administration**	**Frequency**
1. Injectable (medication that is injected into the muscle (intramuscular) or just under the skin (subcutaneous))	1. Daily
2. Pill	2. Weekly
3. Infusion (medication is delivered through a needle placed directly into a vein	3. Monthly

ALL QUALIFIED RESPONDENTS (S100r1)

Q818 If there were a pharmaceutical treatment available for NASH, what would you like for it to do? In other words, what would you consider successful treatment of NASH?

Please select all that apply.

[RANDOM, MULTI-SELECT]

1. Improvement in LFTs/bloodwork
2. Improvement in symptoms (if applicable)
3. Reduction in weight
4. Less frequent visits to the doctor for check-ups
5. NASH biomarker panel normalization
6. Reverting (decrease in severity) my NASH
7. Resolving (getting rid of) my NASH
8. Prevention of cirrhosis
9. Not needing a liver transplant
10. Other, please specify: [INSERT TEXT BOX. ANCHOR]

SECTION 100: DEMOGRAPHICS

ALL QUALIFIED RESPONDENTS (S100r1)
Q103 What is the highest degree you received?

1. High school diploma or the equivalent (GED)
2. Associate degree
3. Bachelor's degree
4. Master's degree
5. Professional degree (MD, DDS, DVM, LLB, JD, DD)
6. Doctorate degree (Ph.D. or Ed.D.)

97. None of the above

ALL QUALIFIED RESPONDENTS (S100r1)
Q105 What best describes your current employment status?

1. Employed full-time
2. Employed part-time
3. A homemaker
4. A full-time student
5. Retired
6. Unable to work for health reasons
7. Furloughed or laid off due to Covid-19
8. Unemployed

9. Other

ALL QUALIFIED RESPONDENTS (S100r1)
Q110 In 2019, what was your household's total yearly income before taxes?

Please remember that your individual information will never be shared. These questions are only used to ensure a representative mix of respondents is achieved.

1. Under $25,000
2. $25,000 - $49,999
3. $50,000 - $74,999
4. $75,000 - $99,999
5. $100,000 - $149,999
6. $150,000 or more
7. Decline to answer

ALL QUALIFIED RESPONDENTS (S100r1)
Q115 Are you currently covered by any of the following types of health insurance or health coverage plans?

Please mark "yes" or "no" for each type of coverage.

[COLUMNS]
1. Yes
2. No

[ROWS]
1. Insurance through a current or former employer or union
2. Insurance purchased directly from an insurance company
3. Medicare, for people 65 and older, or people with certain disabilities
4. Medicaid, Medical Assistance, or any kind of government-assistance plan for those with low incomes or a disability
5. TRICARE or other military healthcare
6. VA (including those who have ever used or enrolled for VA health care)
7. Indian Health Service
8. Any other type of health insurance or health coverage plan

[IF HAS HEALTH INSURANCE (Q115r1-8c1), ASK Q165. ALL OTHERS JUMP TO CLOSING.]

HAS HEALTH INSURANCE (Q115r1-8c1)
Q165 Do you currently have a health insurance plan that helps pay for the cost of prescription drugs?

1. Yes

2. No
3. Not sure

ALL QUALIFIED RESPONDENTS (S100r1)
Q170 Which best describes where you live?

5. Urban
6. Suburban
7. Rural
